# The Dual Interactions of p53 with MDM2 and p300: Implications for the Design of MDM2 Inhibitors

**DOI:** 10.3390/ijms20235996

**Published:** 2019-11-28

**Authors:** Srinivasaraghavan Kannan, Anthony W. Partridge, David P. Lane, Chandra S. Verma

**Affiliations:** 1 Bioinformatics Institute, Agency for Science, Technology and Research (A*STAR), 30 Biopolis Street, #07-01 Matrix, Singapore 138671, Singapore; 2 MSD International, Translation Medicine Research Centre, Singapore 138665, Singapore; anthony_partridge@merck.com; 3 p53 Laboratory, Agency for Science, Technology and Research (A*STAR), 8A Biomedical Grove, #06-04/05, Neuros/Immunos, Singapore 138648, Singapore; dplane@p53lab.a-star.edu.sg; 4 School of Biological Sciences, Nanyang Technological University, 60 Nanyang Drive, Singapore 637551, Singapore; 5 Department of Biological Sciences, National University of Singapore, 14 Science Drive 4, Singapore 117543, Singapore

**Keywords:** stapled peptides, PPIs, MD simulations, phosphorylation, off-target effects

## Abstract

Proteins that limit the activity of the tumour suppressor protein p53 are increasingly being targeted for inhibition in a variety of cancers. In addition to the development of small molecules, there has been interest in developing constrained (stapled) peptide inhibitors. A stapled peptide ALRN_6924 that activates p53 by preventing its interaction with its negative regulator Mdm2 has entered clinical trials. This stapled peptide mimics the interaction of p53 with Mdm2. The chances that this peptide could bind to other proteins that may also interact with the Mdm2-binding region of p53 are high; one such protein is the CREB binding protein (CBP)/p300. It has been established that phosphorylated p53 is released from Mdm2 and binds to p300, orchestrating the transcriptional program. We investigate whether molecules such as ALRN_6924 would bind to p300 and, to do so, we used molecular simulations to explore the binding of ATSP_7041, which is an analogue of ALRN_6924. Our study shows that ATSP_7041 preferentially binds to Mdm2 over p300; however, upon phosphorylation, it appears to have a higher affinity for p300. This could result in attenuation of the amount of free p300 available for interacting with p53, and hence reduce its transcriptional efficacy. Our study highlights the importance of assessing off-target effects of peptide inhibitors, particularly guided by the understanding of the networks of protein-protein interactions (PPIs) that are being targeted.

## 1. Introduction

The tumour suppressor protein p53 is a transcription factor that plays pivotal roles in numerous biological processes, including cell cycle arrest, apoptosis, senescence, and DNA repair [1,2]; its deregulation results in several diseases, and hence it is a major therapeutic target [3,4]. Called the ‘guardian of the genome’ [5], it coordinates cellular responses to various stress signals. Impairment of p53 function is caused by either mutations in the *TP53* gene or overexpression of proteins that control its levels, such as Mdm2 and Mdmx [6].

Mutations in the p53 pathway are implicated in most human cancers [7]. The DNA binding domain of p53 harbors most of the deleterious p53 mutations resulting in impairment of DNA binding or destabilization of the fold of p53 [7]. Therapies are being pursued to restabilize misfolded p53 or to abrogate the interaction of wild type p53 with negative regulators such as Mdm2 and/or Mdmx, which can be overexpressed [6,8,9,10]. They are both E3 ligase components that work with other components of the ubiquitin pathway to target p53 for ubiquitin modifications and proteasome mediated degradation. A major effort targeting Mdm2/Mdmx for inhibition by small molecules and peptides is ongoing in several laboratories and companies [11].

Upon the sensing of stress by a cell, key post-translational events are initiated, particularly those that activate p53. This results in the release of p53 from sequestration by Mdm2/Mdmx, notably by phosphorylation of both Mdm2/Mdmx and p53 [12,13]. The next step in the activation of p53 towards its initiation of the relevant transcriptional programs is its recruitment to the histone acetyltransferase coactivators CREB binding protein (CBP) and p300, which (a) promote local chromatin unwinding [14,15] and (b) acetylate p53 on six C-terminal lysine residues further stabilizing it [16,17,18]. p300 - is a transcriptional co-activator that interacts with the intrinsically disordered transactivation domains of several transcription factors, including p53 [14,19,20,21,22]. 

p300 is composed of seven distinct domains including two transcriptional adaptor zinc-binding (Taz) domains, Taz1 (C/H1) and Taz2 (C/H3), which mediate key protein-protein interactions (PPIs) regulating co-activation. These domains are also known to interact with the N-terminal transactivation domain (TAD) of p53 [23,24]. The p53_TAD can be divided into two subdomains, TAD1 composed of residues 140 and TAD2 composed of residues 41–61, which can independently activate transcription [25]. TAD1 and TAD2 have been shown to interact with both Taz1 and Taz2 of p300 [26,27,28].

Interaction of chromatin-bound p53 with p300 results in acetylation of histones, which facilitates transcription [29], and this is dependent on the amount of p300 binding by p53 [15]. Inhibition of binding by competitor proteins or down-regulation of CBP or p300 by siRNA has been found to result in reduction in local histone acetylation and p53-mediated transcription [15,30,31,32]. The importance of the interaction between p53 and the Taz2 domain of p300 was underscored by the observation that catalytically-inactive deletion mutants of p300 containing this domain can inhibit p53-dependent apoptosis and G1 arrest [23,33]. 

The direct interaction between p53 and p300 complex was demonstrated by NMR spectroscopy [34,35,36,37,38,39]. p53 forms a short α-helical conformation within residues 17–26 in complex with Taz2. The complex is stabilized by hydrophobic and specific electrostatic interactions. p53_TAD is intrinsically disordered [40] and characterized by great conformational flexibility in solution, and thus easily participates in numerous interactions with diverse proteins [41]. The p53_TAD1 peptides are known to form short (for example, residues 17–26) amphipathic helices in complex with proteins such as p300_Taz2, Mdm2, and Mdmx. It is the same region of p53 that interacts with both Mdm2/Mdmx and p300_Taz2, and while there are differences in specific interactions, hydrophobicity is thought to be the main driver in these associations. This led us to wonder whether inhibitors designed against Mdm2 to release p53 may also interact with the p53-binding region of p300_Taz2, thus attenuating the effects of p53 and, if so, could a negative feature be designed into the inhibitors to prevent them from binding to p300_Taz2. 

In particular, with the recent development of novel therapeutics targeting Mdm2/Mdmx [42,43,44], notably stapled peptides, these designs may result in improved therapeutic efficacy. We present here a study investigating the binding of the p53 peptide and stapled peptide inhibitors of Mdm2/Mdmx with p300 using computational approaches.

## 2. Results

### 2.1. p53_TAD1 Binding with Mdm2

**Figure 1:**
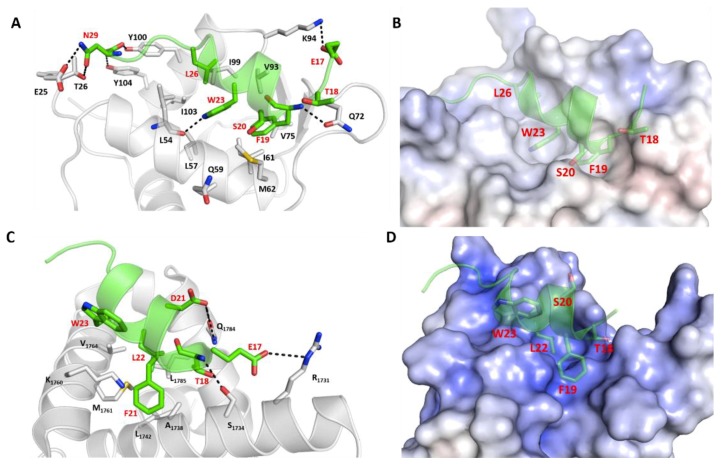
Structural and energetic basis for the binding of p53_TAD1 peptide with Mdm2 and p300. Cartoon/surface representations, based on the crystal and NMR structures, depicting how p53_TAD1 (residues 17–29) interacts with (**A**,**B**) the N-terminal domain (residues 25–109) of Mdm2 (**C**,**D**) p300 protein (residues 1723–1812). (**A**,**C**) the p53_TAD1 peptide is shown as the green cartoon with the receptor protein shown as the grey cartoon. Residues in the binding pocket are highlighted and the protein-peptide H-bond interactions are highlighted (dashed lines). (**B**,**D**) the p53_TAD1 peptide is shown as the green cartoon with the bound proteins shown with their electrostatic potential surfaces (red and blue correspond to–5 kcal/mol and 5 kcal/mol potentials, respectively).

The N-terminal transactivation domain (TAD1) of p53 (p53_TAD1), highly flexible in solution, adopts an alpha helical conformation from residues 17–29 when bound to a largely hydrophobic pocket in the N-terminal domain of Mdm2 and Mdmx (Figure 1A,B). The p53_TAD1 peptide adopts an amphipathic α-helical conformation with the side chains of Phe19, Trp23, and Leu26 buried inside the hydrophobic pocket of Mdm2/Mdmx. In addition to the hydrophobic interactions, intermolecular hydrogen bonds (H-bonds) between the side chain of p53_TAD1 residue Glu17 with the side chain of Lys94 from Mdm2, the backbone of p53_TAD1 residue Phe19 with the side chain of Gln72 from Mdm2, the side chain of p53_TAD1 residue Trp23 with the backbone of Leu54 from Mdm2, the backbone of p53_TAD1 residue Asn29 with the side chain of Tyr104 from Mdm2, and the side chain of Asn29 with the side chain of Glu25 and Thr26 from Mdm2 (Figure 1A) stabilize the complex.

**Figure 2:**
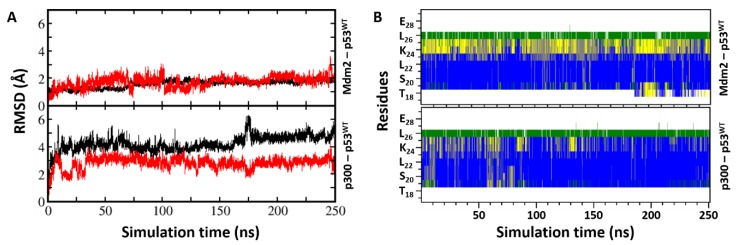
(**A**) The structural changes that occur in the p53_TAD1 peptide and in Mdm2 or in p300 during the molecular dynamics (MD) simulations of the complexes as measured by root mean squared deviation (RMSD). The RMSD of the peptide (calculated against the starting conformation of the MD simulations) is shown in red for the Mdm2 complex (top of panel **A**) and for the p300 complex (bottom of panel **A**); the RMSD of the protein (calculated against the starting conformation of the MD simulations) is shown in black for the Mdm2 complex (top of panel **A**) and for the p300 complex (bottom of panel **A**). Panel B shows the changes in the secondary structures of the p53_TAD1 peptides when bound to Mdm2 (top of panel **B**) or p300 (bottom of panel **B**); the secondary structures were calculated using the DSSP program and are shown as follows: blue for α-helix, grey for 3_10_-helix, yellow for turn, green for bend, and white for coil, along the peptide chain (y-axis) as a function of the simulation time (x-axis). The graphs show that the simulations are stable.

Molecular dynamics (MD) simulations of the p53_TAD1-Mdm2 complex remained stable and close to the experimental conformation, a feature reported in numerous studies [45]. The root mean squared deviations (RMSDs) of the bound p53_TAD1 peptide and Mdm2 do not deviate much in their conformations, remaining within ~2.5 Å (Figure 2) from the starting conformations. The side chains of Phe19, Trp23, and Leu26, all buried inside a hydrophobic cavity on the surface of Mdm2, and the intermolecular H-bonds between the side chain of p53_TAD1 residue Glu17 with the side chain of Lys94 from Mdm2, the backbone of p53_TAD1 residue Phe19 with the side chain of Gln72 from Mdm2, the side chain of p53_TAD1 residue Trp23 with the backbone of Leu54 from Mdm2, and the backbone of p53_TAD1 residue Asn29 with the side chain of Tyr104 from Mdm2 were, all retained (~75% of the simulation time).

### 2.2. p53_TAD1 Binding with p300_Taz2

The solution structure of p53_TAD1–p300_Taz2 complex shows the p300_Taz2 domain as constituted by four core α-helices [46]. The p53_TAD1 peptide (1–39) forms a short α-helical conformation within residues 17–26 in complex with p300_Taz2, while the rest of the p53_TAD1 peptide is unfolded and exposed to the solvent (Figure S1). In contrast to the hydrophobic p53 peptide pocket in Mdm2, the p53 binding pocket in p300_Taz2 is relatively positively charged (Figure 1D). Phe19 of p53_TAD1 is buried into the hydrophobic pocket made up of residues Ala1738, Leu1742, and Met1761. The side chain of Leu22 from p53_TAD1 peptide is buried into the hydrophobic pocket made up of residues Met1761, Val1764, and Leu1785. Electrostatic interactions are also key at the p53_TAD1-p300_Taz2 interface. The p53_TAD1 binding site of p300_Taz2 has positively charged regions (Figure 1C,D) with a salt bridge between Glu17 of p53_TAD1 and Arg1731 of p300_Taz2. Interactions between the side chains of Asp21 from p53_TAD1 and Gln1784 of p300_Taz2 and interactions between the carbonyl backbone of Thr18 from p53_TAD1 and the hydroxyl group on the side chain of Ser1734 from p300_Taz2 further stabilize the complex.

We explored the stability of two complexes of p300_Taz2 with p53_TAD1: residues 1–39 as has been resolved in its complex with p300_Taz2 by NMR and residues 17–29, having deleted the remainder from the solution structure of p53_TAD1(1–39)–p300_Taz2. During the simulation of the p53_TAD1 (1–39)–p300_Taz2 complex, the bound peptide exhibited high flexibility without adopting any particular secondary structure, particularly the regions of p53_TAD1 from residues 1–17 and 30–39, which were shown not to adopt any defined secondary structures in the NMR ensembles and remain flexible, and did not adopt any secondary structures (Figure S1); however, residues 17–29 remain alpha helical. In contrast, during the simulation of the p53_TAD1(17–29)–p300_Taz2 complex, the bound peptide does not deviate much in its conformations with RMSD of sampled conformations within ~3.0 Å (Figure 2) from its bound state, and remain alpha helical (Figure 2). Therefore, we decided to focus only on the p53_TAD1(17–29)–p300_Taz2 complexes as this length of p53 is also the length of the peptides that have been designed as inhibitors of Mdm2/Mdmx. During the simulation, the p53_TAD1 peptide bound structure of p300_Taz2 remains stably bound with RMSD of sampled conformations within ~4 Å. The binding of the shorter p53_TAD1 peptide (17–29) is stabilized by both hydrophobic and H-bond interactions. Hydrophobic residues Phe19, Leu22, and Leu26 all remain buried in the hydrophobic pocket of p300_Taz2. The negatively charged residues at the termini of p53_TAD1 interact with the positively charged residues from p300_Taz2 (side chain of p53_TAD1–Glu17 with the side chain of p300_Taz2–Arg1731 and side chain of p300_Taz2–Arg1732; side chain of p53_TAD1–Asn29 with the side chain of p300_Taz2–Arg1763; backbone of p53_TAD1–Pro27 with the side chain of p300_Taz2–His1767). In addition, the side chain of p53_TAD1–Thr18 and p53_TAD1–Ser20 are in close proximity to the positively charged regions of the p300_Taz2 pocket (Figure 1D and Figure S2).

### 2.3. Comparison of p53_TAD1 Binding with Mdm2 and p300_Taz2

It is clear that the intrinsically disordered N-terminal TAD1 region (residues 17–29) of p53 adopts an alpha helical conformation upon binding to either Mdm2 or p300_Taz2, although the binding pocket in both proteins have somewhat differing physicochemical properties, with one pocket being highly hydrophobic and the other positively charged (Figure 1B,D). Despite this difference, the p53_TAD1 peptide adopts similar conformations and utilizes an overlapping set of residues to interact with these two proteins. The p53_TAD1 peptide binds to Mdm2 with a higher affinity (ΔG~ –62 kcal/mol) compared with p300_Taz2 (ΔG ~ –56 kcal/mol) (Figure S3); this is in qualitative agreement with experimental studies; Feng et al. [36] reported that the p53_TAD1 peptide (residues 1 to 39) binds to Mdm2 with Kd ~400 nM and to p300 with Kd ~3 μM. The binding of p53_TAD1 to Mdm2 is largely driven by bulky hydrophobic residues Phe19, Trp23, and Leu26. These hydrophobic residues also contribute to the binding to p300_Taz2; however, their contribution is smaller by ~2 kcal/mol (Figure S3) and, in addition, there are significant contributions from Asp21, Leu22, Leu25, and Asn29 for the binding to p300_Taz2.

### 2.4. Effect of Phosphorylation on the Binding of Peptides with p300_Taz2 and Mdm2

**Figure 3:**
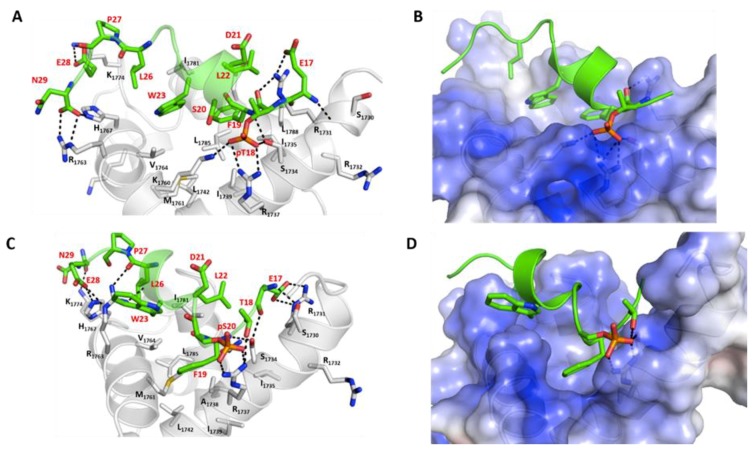
Structural and energetic basis for the binding of the phosphorylated p53_TAD1 peptide and p300. Cartoon/surface representations, based on a representative conformation extracted from the MD simulations, of the p53_TAD1 peptide phosphorylated at Thr18 (panel **A** showing the structural details; panel **B** showing the electrostatic potentials) bound to p300 and of the p53_TAD1 peptide phosphorylated at Ser20 bound to p300 (panel **C** showing the structural details; panel **D** showing the electrostatic potentials). In panels A and C, the phosphorylated p53_TAD1 peptide is shown as the green cartoon and the protein is shown as the grey cartoon; residues of the peptides and the binding pocket of p300 are highlighted as sticks and the protein–peptide H-bond interactions are highlighted as dashed lines. In panels B and D, the phosphorylated p53_TAD1 peptide is shown as the green cartoon and p300 is shown with its electrostatic potential surface (red and blue correspond to–5 kcal/mol and 5 kcal/mol potentials, respectively). The figure highlights the observation together with the multiple H-bonds, the anionic phosphorylated residues are also stabilized by a strong cationic surface of p300.

**Figure 4:**
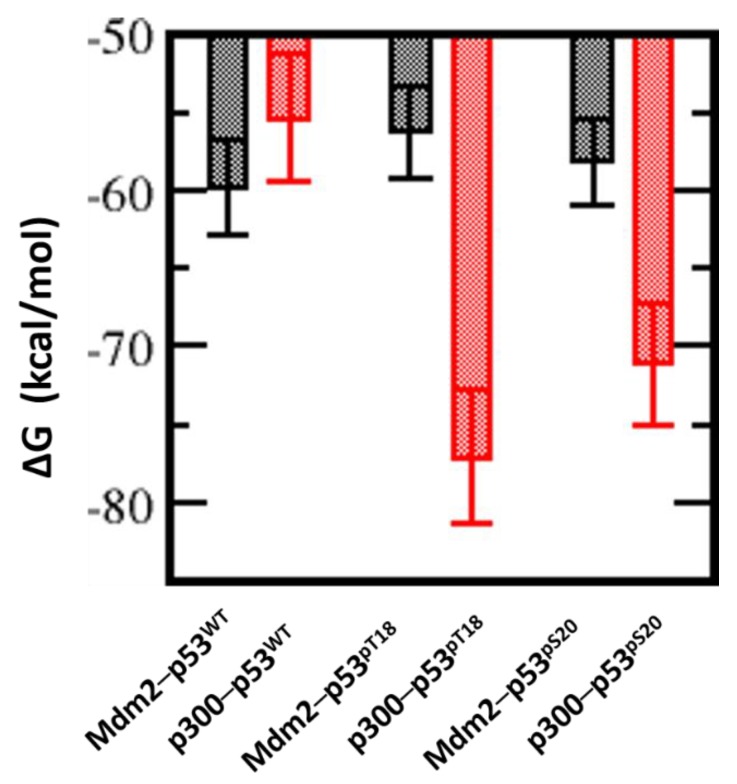
Estimation of the free energies (ΔG) of the interactions between the p53_TAD1 peptides (unphosphorylated and phosphorylated) and Mdm2 or p300, using the molecular mechanics Poisson Boltzmann surface area (MMPBSA) approximations from the conformations generated from MD simulations of the complexes. Higher affinities are reflected by larger negative values; it is clear that the unphosphorylated p53_TAD1 peptide–Mdm2 interactions are of a higher affinity than the unphosphorylated p53_TAD1 peptide–p300 interactions; this trend reverses when the peptides are phosphorylated.

Although the p53_TAD1 peptide binds to p300_Taz2 with lower affinity compared with its binding to Mdm2, phosphorylation of p53, particularly of Thr18 and Ser20, is known to enhance its binding to p300_Taz2; phosphorylation of Thr18 (pThr18) is known to increase its affinity for p300_Taz2 by 11-fold as compared with the unphosphorylated form (0.2 µM versus 3.0 µM) [39]. At the same time, phosphorylation of Thr18 results in a reduction in the affinity of p53_TAD1 for Mdm2 (1.3 µM versus 0.2 µM) [39]. In contrast, phosphorylation of Ser20 (pSer20) is known to not affect the affinity of p53 for Mdm2. Molecular dynamics (MD) simulations of the complex between pThr18_p53_TAD1 and p300_Taz2 show that the bound peptide remains stably bound with RMSD ~3 Å (Figure S4) against the starting conformation. However, the bound alpha helical conformation is less preserved (Figure S4) as the presence of additional phosphate groups enhances the flexibility of the peptides even in the bound states. The bound peptide retained all the H-bond (side chain of p53_TAD1–Glu17 with the side chain of p300_Taz2–Arg1731; backbone of p53_TAD1–pT18 with the side chain of p300_Taz2–Arg1731; side chain of p53_TAD1–Asn29 with the side chain of p300_Taz2–Arg1763; and side chain of p53_TAD1–Glu28 with the side chain of p300_Taz2–Lys1760) and hydrophobic interactions (Phe19, Leu22, and Leu26 all remain buried) observed between p53_TAD1 and p300 (Figure 3). In addition, phosphorylated pThr18 is engaged in charge-charge interactions with the side chains of Ser1734, Arg1732, and Lys1763 (Figure 3). This additional charge-charge interaction between the negatively charged pThr18 from p53_TAD1 (phosphorylated peptide) and the positively charged p300_Taz2 results in increased binding between the two (Figure 3). In agreement with the experimental observations, our binding energy calculations show an energy of ~ –77 kcal/mol (Figure 4) for pThr18_p53_TAD1 binding to p300 compared with ~ –56 kcal/mol for the binding of unphosphorylated p53_TAD1 to p300.

In the case of the complex between pSer20_p53_TAD1 and p300_Taz2, the bound peptide remains stably bound with RMSD ~3 Å (Figure S4) against the starting conformation. However, the bound alpha helical conformation is less preserved (Figure S4). The side chain of hydrophobic residues Phe19 and Leu26 of the bound peptide remain buried in the hydrophobic pocket of p300_Taz2. In comparison with the p53_TAD1–p300_Taz2 interactions, the side chain of peptide residue Glu17 retains its interaction with the side chain of Arg1731 from p300_Taz2 and forms additional interactions with the side chain of Ser1730 from p300_Taz2. The side chain of p53_TAD1 Asn29 loses its interaction with Arg1763 from p300_Taz2; however, this loss is compensated for by the formation of new interactions between the peptide residue Asn29 with His1767 and Lys1774 from p300_Taz2 (Figure 3). The p300_Taz2 residue Arg1763 interacts with the backbone of peptide residues Pro27 and Leu26 (Figure 3). In addition, the phosphorylated pSer20 forms interactions with the side chains of Arg1737 and Ser1734 from p300_Taz2. These additional charge-charge interactions are expected to contribute towards improved binding between the pSer20_p53_TAD1 and p300_Taz2. Our binding energy calculations show that the associated binding energy is ~ –71 kcal/mol compared with the energy of ~ –56 kcal/mol for the binding of p53_TAD1 and p300_Taz2 (Figure 4).

Thus, it is clear that both phosphorylated forms of the p53_TAD1 peptide (pThr18 and pSer20) show improved binding to p300_Taz2 compared with the unphosphorylated p53_TAD1, largely through the formation of new charge-charge interactions. To understand the structural basis for the observed energetic difference between the peptides, we carried out detailed structural analysis of the complex trajectories. In the unphosphorylated form of the bound p53_TAD1 peptide, the side chain of the Thr18 is facing the positively charged surface of p300_Taz2. In contrast, the side chain of Ser20 is exposed to the solvent. Upon phosphorylation, the negatively charged pSer20 forms charge-charge interactions with the side chains of Arg1737 and Ser1734 from p300_Taz2. This is accompanied by partial unfolding of a helical turn at the N-terminus of the peptide. Given that the Ser20 is located between the Phe19 and Trp23 of the p53 peptide, which is known to retain its helicity even in its unbound state, conformational changes induced by the formation of new charge-charge interactions do not contribute favourably to the interactions. On the other hand, Thr18, which is located at the N- terminal end of the peptide, can undergo conformational rearrangements to form new interactions, without affecting the bound conformation of the rest of the peptide. Therefore, the addition of phosphate groups in both cases (pThr18 and pSer20) probably contribute favourably towards the observed increases in binding; however, the improved binding is offset partially in the case of pSer20 by the conformational constraints imposed by its location in the peptide (Figure 3 and Figure 4).

How does phosphorylation affect binding to Mdm2? We have seen above that the parent p53_TAD1 peptide displayed higher affinity for Mdm2 compared with p300_Taz2. Mdm2 is defined by a surface that is highly hydrophobic with small clusters of positively and negatively charged regions scattered. In contrast, p300_Taz2 protein, which has a positively charged surface, immediately suggesting a higher affinity for the negatively charged phosphorylated peptides. In the crystal structure of the p53_TAD1–Mdm2 complex, Thr18 from p53_TAD1 is in the proximity of a negatively charged region on Mdm2 with the side chain of Thr18 from p53_TAD1, facing and engaging in interactions with the backbone and side chain of Asp21 from p53_TAD1. In contrast, Ser20 points towards a region of Mdm2 that is slightly positively charged. The side chain of Ser20 points towards the side chain of Gln59 and Met62 from Mdm2, but makes no specific interactions (Figure 1A). 

**Figure 5:**
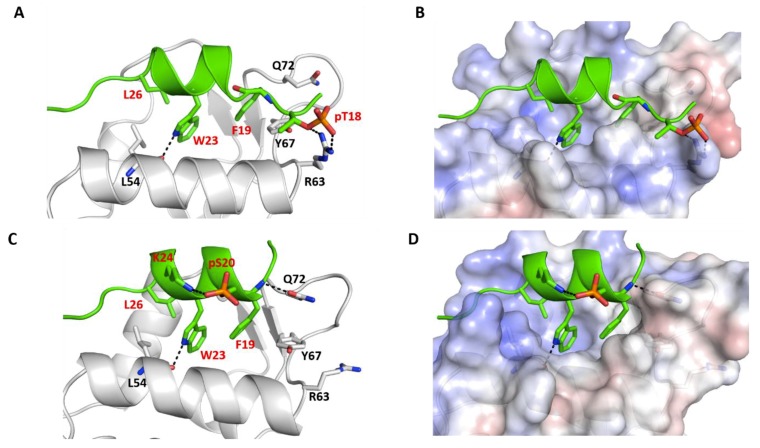
Structural and energetic basis for the binding of the phosphorylated p53_TAD1 peptide and Mdm2. Cartoon/surface representations, based on a representative conformation extracted from the MD simulations, of the p53_TAD1 peptide phosphorylated at Thr18 (panel **A** showing the structural details; panel **B** showing the electrostatic potentials) bound to Mdm2 and of the p53_TAD1 peptide phosphorylated at Ser20 bound to Mdm2 (panel **C** showing the structural details; panel **D** showing the electrostatic potentials). In panels A and C, the phosphorylated p53 _TAD1 peptide is shown as the green cartoon and the protein is shown as the grey cartoon; residues of the peptides and the binding pocket of Mdm2 are highlighted as sticks and the protein–peptide H-bond interactions are highlighted as dashed lines. In panels B and D, the phosphorylated p53_TAD1 peptide is shown as the green cartoon and Mdm2 is shown with its electrostatic potential surface (red and blue correspond to –5 kcal/mol and 5 kcal/mol potentials, respectively). The figure highlights the observation that together with the multiple H-bonds, the anionic phosphorylated residues are also stabilized by a cationic surface of Mdm2, which is not as strongly cationic as that of p300.

During the simulation of pThr18_p53_TAD1-Mdm2, the bound peptide showed increased flexibility (compared with the unphosphorylated form), with the RMSD of the bound peptide reaching up to 3 Å with respect to its starting structure (Figure S5). In addition, we notice that only the side chain of Trp23 remains buried in the hydrophobic pocket on the Mdm2 surface, while those of Phe19 and Leu26 are constantly fluctuating (Figure 5). In addition, out of several H-bond interactions (between the side chain of p53_TAD1 residue Glu17 and the side chain of Lys94 from Mdm2, the backbone of p53_TAD1 residue Phe19 and the side chain of Gln72 from Mdm2, the side chain of p53_TAD1 residue Trp23 and the backbone of Leu54 from Mdm2, the backbone of p53_TAD1 residue Asn29 and the side chain of Tyr104 from Mdm2) observed in the p53_TAD1–Mdm2 simulations, only one H-bond interaction (side chain of p53_TAD1 residue Trp23 with backbone of Leu54 from Mdm2) was retained, (~40% of simulation time), with the other H-bonds lasting for less than 5% of the simulation time. It is clear that the addition of a phosphate group onto the side chain of Thr18 is not well tolerated structurally (partially owing to its location in the vicinity of the negatively charged Mdm2 surface and partly from repulsion from the spatially contiguous negatively charged Asp21 side chain) (Figure 5). The side chain of Thr18 is involved in H-bond interactions with the backbone of Asp21 and is important for retaining the helical turn at the N-terminus of the bound peptide. Addition of the phosphate group in Thr18 results in loss of this key H-bond interaction with the Asp21 backbone, thus resulting in loss of alpha helicity in the pThr18_p53_TAD1 peptide, even when it is bound to Mdm2 (Figure 5A and Figure S5). The presence of additional negative charges on the Mdm2 surface further act to repel the negative charge on pThr18 and the phosphorylated Thr18 undergoes conformational rearrangements to form new interactions (Figure 5). One such interaction is with the side chain of Arg63 from Mdm2; however, this interaction is very transient in our simulations. The loss of interactions between the pThr18_p53_TAD1 peptide with Mdm2 is reflected in binding energy calculations, as the binding free energy of the phosphorylated peptide (pThr18_p53_TAD1) to Mdm2 is decreased by ~4 kcal/mol as compared with the binding of unphosphorylated p53_TAD1 peptide (~ –56 kcal/mol versus ~ –60 kcal/mol for the phosphorylated and unphosphorylated peptide, respectively) (Figure 4). The predicted loss in affinity is in good qualitative agreement with experimental data, with a seven-fold loss in affinity for the phosphorylated peptide as compared with its unphosphorylated form, with Kd of 1.3 µM and 0.2 µM for the phosphorylated and unphosphorylated peptides respectively, and with previous simulation data [47,48]. 

In contrast, during the simulation of the peptide pSer20_p53_TAD1 bound to Mdm2, the bound peptide remained stably bound, with RMSD ~1.5 Å relative to its starting structure, and exhibited fluctuations similar to the parent unphosphorylated peptide (RMSD of ~1 Å) (Figure S5). The side chains of the key hydrophobic residues Phe19, Trp23, and Leu26 all remain buried in the hydrophobic pocket of the Mdm2 surface, as was exhibited in the unphosphorylated complex. In addition, several H-bond interactions (between the backbone of p53_TAD1 residue Phe19 and the side chain of Gln72 from Mdm2, the side chain of p53_TAD1 residue Trp23 and the backbone of Leu54 from Mdm2) observed in the p53_TAD1–Mdm2 simulations were also retained (~90%) during the simulation of the pSer20_p53_TAD1–Mdm2 complex (Figure 5). The addition of negatively charged phosphate groups onto the side chain of Ser20, which faces the Mdm2 surface, but without engaging in any specific interactions with Mdm2 or itself, is well tolerated structurally. Upon phosphorylation, the side chain of pSer20 interacts with the side chain of Lys24 from p53_TAD1, which in turn is not involved in any specific interactions with other sites on Mdm2. This additional interaction adds to the structural stability of the bound peptide. Our binding energy calculations reflect our structural analysis, which shows that the pSer20 peptide (pSer20_p53_TAD1) binds with affinity (~ –60 kcal/mol) similar to that of the p53_TAD1 peptide (~ –59 kcal/mol) (Figure 4). This is in good agreement with the experimental data, which showed that both the Ser20 phosphorylated and unphosphorylated p53_TAD1 peptides bind Mdm2 with similar affinities (Kd of 0.17 µM and 0.2 µM for the phosphorylated and unphosphorylated peptides, respectively).

### 2.5. Effect of Phosphorylation on the Structural Dynamics of p53 Peptide

From our simulation, we observe that the phosphorylation affects the binding of peptides with its interaction partners. We next investigated how the phosphorylation affects the free peptide in solution. During the biasing potential replica exchange molecular dynamics (BP-REMD) simulation, the p53WT peptide is highly flexible and remains mostly unstructured in solution, with only ~11% of sampled conformations adopting alpha helicity (Figure S6). Introduction of phosphorylation at Thr18 or Ser20 does not enhance conformational rigidity of the free peptide; rather, a slight decrease in structural ordering was observed during MD simulations. Only ~8% of sampled structures adopt helical conformations in the case of pThr18_p53_TAD1 and pSer20_p53_TAD1 peptides (Figure S6). Although peptide phosphorylation is known to induce large conformation changes in peptides inducing disorder to order conformations (vice versa), no major change observed in our simulations suggests that, in the case of the p53_TAD1 peptide, pThr18 and pSer20 have no major effect on the conformation of the free peptide. Given that the unbound conformations of these peptides are very similar, but adopt alpha helical conformation upon binding to Mdm2, the entropic contribution (the entropic energy for the disorder to order transition upon binding) for binding to Mdm2 is expected to be similar for all these peptides. Therefore, the difference in binding energies observed for the WT_p53_TAD1, pThr18_p53_TAD1, and pSer20_p53_TAD1 peptides with Mdm2 is mostly enthalpic.

### 2.6. Structure and Dynamics of ATSP_7041–Mdm2 and ATSP_7041–p300 Complexes

**Figure 6:**
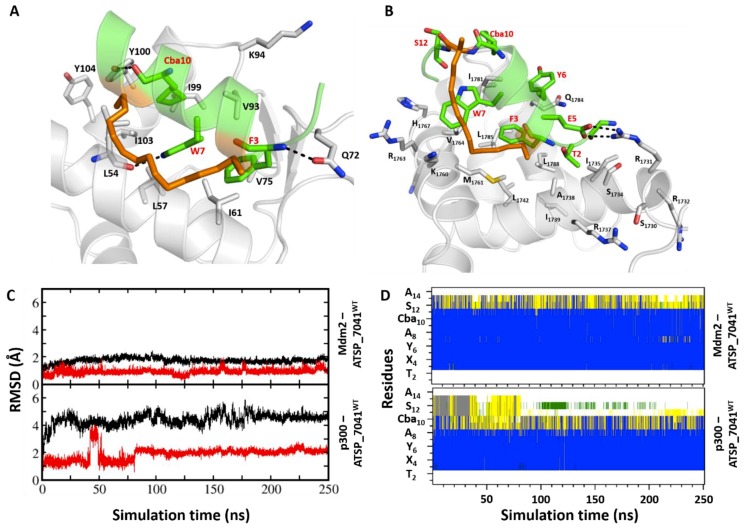
Structural basis for the binding of the stapled peptide ATSP_7041 peptide with Mdm2 and p300. Cartoon representations (panels **A** and **B**), based on a representative conformation extracted from the MD simulations of ATSP_7041 bound to Mdm2 and bound to p300, respectively; the ATSP_7041 peptide is shown as the green cartoon with the receptor protein is shown as the grey cartoon. Residues in the binding pocket are highlighted and the protein–peptide H-bond interactions are highlighted (dashed lines); the staple (hydrocarbon linker) is shown as orange sticks. It is clear that the peptide is stabilized by multiple H-bond and packing interactions; the staple derives affinity by interacting with the surface of the proteins. Panels C and D show the structural changes that occur in the stapled peptide ATSP_7041 when bound to Mdm2 or to p300, as measured by RMSD and secondary structures. The RMSD of the peptide (calculated against the starting conformation of the MD simulations) is shown in red for the Mdm2 complex (top of panel **C**) and for the p300 complex (bottom of panel **C**); the RMSD of the protein (calculated against the starting conformation of the MD simulations) is shown in black for the Mdm2 complex (top of panel **C**) and for the p300 complex (bottom of panel **C**). Panel D shows the changes in the secondary structures of the stapled peptide ATSP_7041 when bound to Mdm2 (top of panel **D**) or p300 (bottom of panel **D**); the secondary structures were calculated using the DSSP program and are shown as follows: blue for α-helix, grey for 3_10_-helix, yellow for turn, green for bend, and white for coil, along the peptide chain (y-axis) as a function of the simulation time (x-axis). The graphs show that the simulations are stable.

With this observation of peptides sharing binding characteristics with both Mdm2/Mdmx and p300, we next explore whether a high affinity peptide inhibitor of Mdm2 would also bind to p300. We choose a stapled peptide ATSP_7041 (LTF*R_8_*EYWAQ*S_5_*CbaSAA), which is an analogue of the stapled peptide ALRN_6924, which is currently in clinical trials for oncology [41]. ATSP_7041 was designed successfully with a staple to constrain the peptide to adopt an alpha helical conformation in solution so that reorganization into a helical bound conformation is minimal, thus improving the affinity. This peptide is known to bind to both Mdm2 and Mdmx in a helical conformation, with hydrophobic residue Phe3, Trp7, and Cba10 (Figure 6A, equivalent to Phe19, Trp23, and Leu26 in p53_TAD1) occupying the hydrophobic pocket on the surface of Mdmx and Mdm2, mimicking the binding of the parent p53_TAD1 peptide with a much higher affinity.

We generated a model of the stapled peptide ATSP_7041 bound to Mdm2 based on the available crystal structures of ATSP_7041 bound to Mdmx (PDB id 4N5T). During the MD simulations of this complex, the bound conformation of ATSP_7041 and of Mdm2 remained stable, with RMSD ~2.5 Å against the starting conformation (Figure 6C), and helicity of the peptide well preserved during simulations (Figure 6D). The peptide engages in several H-bonds with the protein, as seen in the original p53_TAD1-Mdm2 structure (Figure 1A) including between the (1) side chain N of Trp7 from peptide and the carbonyl backbone of Leu54 from Mdm2; and (2) backbone N of Phe3 from peptide and the side chain O of Gln72 from Mdm2 (Figure 6A). These are complemented with several transient H-bond interactions involving Lys52, Arg98, and Tyr100 of Mdm2 and the backbone atoms of Ser12, Ala13, and Ala14 of the peptide. In addition, the peptide is also stabilized by hydrophobic interactions mediated by Phe3, Trp7, and Cba10.

We next generated the model of stapled peptide ATSP_7041 bound with p300_Taz2 using the structure of p53_TAD1(17-29) bound to p300_Taz2 (Figure 6B). The bound ATSP-7041 remained stable with RMSD ~2.5 Å (Figure 6C) against the starting conformation, with peptide helicity maintained (Figure 6D) during simulations and p300_Taz2 remaining stable with RMSD ~4.5 Å (Figure 6C). The bound conformation of ATSP_7041 is stabilized by hydrophobic and H-bond interactions with the p300_Taz2. The hydrophobic side chain of Phe3 from ATPS_7041 buried into the hydrophobic pocket of p300_Taz2 occupied by Phe19 from the p53_TAD1 peptide. The side chain of Glu5 from peptide is involved in charge–charge interactions with the side chain of Arg1731 from p300_Taz2 (Figure 6B). The side chain of Trp7 is sandwiched between the hydrophobic residues Val1764, Ile1781 from p300_Taz2, and the hydrocarbon staple linker from the bound ATSP_7041.

The binding energy of ATSP_7041 to Mdm2 is stronger (ΔG ~ –62 kcal/mol) compared with its binding with p300_Taz2 (ΔG ~ –55 kcal/mol), a trend that is similar to the binding of the parent p53_TAD1–peptide (Figure S7). Interestingly, while peptide residues Phe3, Trp7, and Cba10, along with the staple linker, contribute to the Mdm2 binding, only Phe3 contributes the most, along with a reduced contribution from Trp7 among the hydrophobic residues (Figure S7). The staple linkers, which contribute to the binding with Mdm2, make negligible or very little contributions to p300_Taz2 binding. This arises because, in the case of Mdm2, the staple linkers interact with the surface of the Mdm2 binding pocket, while in the case of p300_Taz2, they are exposed to the solvent and not involved in any interactions with p300_Taz2. p300_Taz2 is positively charged in the binding region, and hence Glu5 from ATSP_7041 makes a significant contribution of ~ –6 kcal/mol to the binding, whereas Glu5 makes negligible contributions (~ –1 kcal/mol) to Mdm2 binding (Figure S7).

### 2.7. Effect of Phosphorylation on the Binding of ATSP_7041 with p300 and Mdm2

Given that the phosphorylation of Thr18 and Ser20 both had different effects on binding of p53_TAD1 with p300_Taz2 and Mdm2, we next turned our attention to ATSP_7041. This peptide has a Thr at position 2 (equivalent to Thr18 in the p53_TAD1^WT^), Tyr at position 6, and Ser at position 12. We wondered what the consequences would be if ATSP_7041 were to undergo phosphorylation at these three positions. To investigate this, we carried out MD simulations of the complexes of Thr2, Tyr6, and Ser12 phosphorylated ATSP_7041 with Mdm2 and with p300_Taz2.

**Figure 7:**
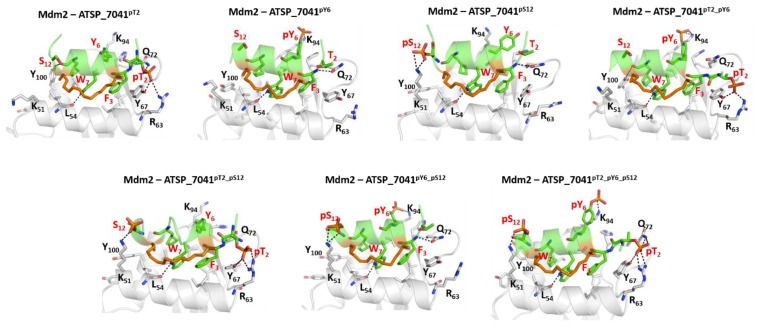
Structural basis for the binding of the phosphorylated stapled peptide ATSP_7041 and Mdm2. Cartoon representations, based on representative conformations extracted from the MD simulations of the ATSP_7041 peptide phosphorylated at Thr2, Tyr6, and Ser12 bound to Mdm2. The phosphorylated ATSP_7041 peptide is shown as the green cartoon and the Mdm2 is shown as the grey cartoon; residues of the peptides and the binding pocket of Mdm2 are highlighted as sticks and the protein-peptide H-bond interactions are highlighted as dashed lines. The figure shows that each phosphate moiety engages in ionic interactions, which further stabilize the ATSP_7041 in its bound states.

**Figure 8:**
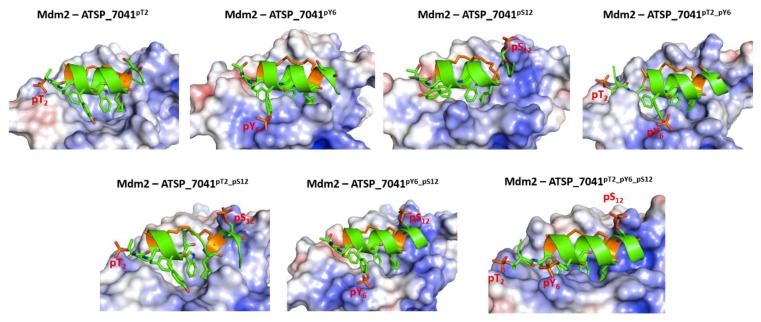
Structural and energetic basis for the binding of the phosphorylated stapled peptide ATSP_7041 peptide and Mdm2. Cartoon/surface representations, based on representative conformations extracted from the MD simulations of the ATSP_7041 peptide phosphorylated at Thr2, Tyr6, and Ser12 bound to Mdm2. The phosphorylated ATSP_7041 peptide is shown as the green cartoon and the Mdm2 is shown as the grey cartoon; Mdm2 is shown with its electrostatic potential surface (red and blue correspond to the –5 kcal/mol and 5 kcal/mol potentials, respectively). It is clear that the cationic surface of Mdm2 attracts the anionic surface of the phosphorylated ATSP_7041.

In the case of the complex with Mdm2, the bound peptides remained stably bound, with RMSD of ~2 Å against the starting conformation (Figure S8). The side chains of the hydrophobic residues Phe3, Trp7, and Cba10 all remained buried in the hydrophobic pocket on the Mdm2 surface. The H-bond interaction (Trp7 side chain with Leu54 backbone) between the bound ATSP_7041 and Mdm2 is retained (90%) throughout the simulations (Figure 7). The H-bond interaction between the side chain of Gln72 and the backbone of Phe3 is preserved only in Mdm2–ATSP_7041^pY6^, Mdm2–ATSP_7041^pS12^, and Mdm2–ATSP_7041^pY6_pS12^ (when Thr2 is unphosphorylated). When Thr2 is phosphoryalted, this Gln72–Phe3 H-bond is lost and the side chain of the phosphorylated pThr2 from ATSP_7041^pT2^, ATSP_7041^pT2_pY6^, ATSP_7041^pT2_pS12^, and ATSP_7041^pT2_pY6_pS12^ interacts with the side chains of Arg63, Tyr67, and Gln72 from Mdm2. In the case of phosphorylation of Tyr6 in ATSP_7041^pY6^, ATSP_7041^pT2_pY6^, ATSP_7041^pY6_pS12^, and ATSP_7041^pT2_pY6_pS12^, the anionic phosphate on the Tyr6 is stabilized (~90% of the simulation) by interactions with the positively charged side chain of Lys94 from Mdm2. Similarly, the negative charge on Ser12 in ATSP_7041^pS12^, ATSP_7041^pY6_pS12^, ATSP_7041^pT2_pS12^, and ATSP_7041^pT2_pY6_pS12^ interacts (~90% of the simulation time) with the positively charged side chain of Lys51 from Mdm2 (Figure 8). The overall bound helical conformation of these phosphorylated peptides was maintained during the simulations of their complexes with Mdm2; some loss in helicity was observed at the N- and C-termini for the phosphorylated Thr2 and phosphorylated Ser12 containing peptides (Figure S9).

**Figure 9:**
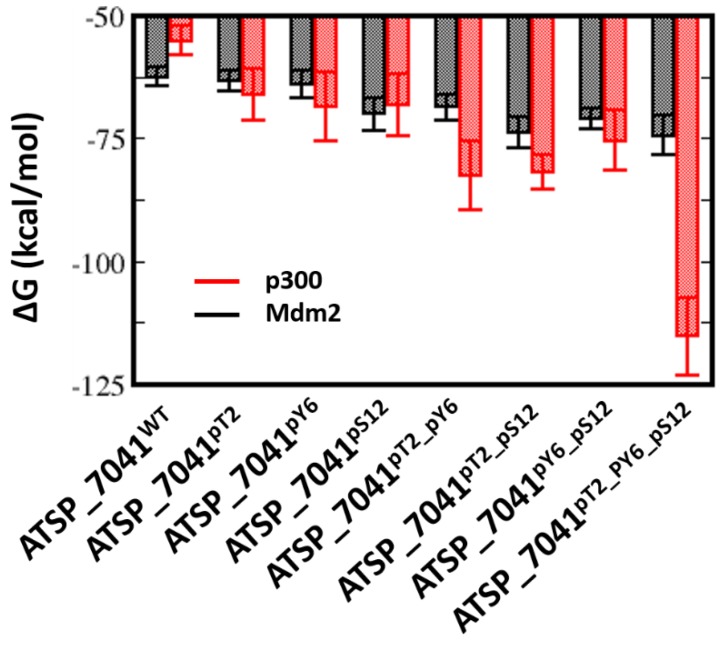
Estimation of the free energies (ΔG) of the interactions between the ATSP_7041 peptides (unphosphorylated and phosphorylated) and Mdm2 or p300, using the MMPBSA approximations from the conformations generated from MD simulations of the complexes. Higher affinities are reflected by larger negative values; it is clear that the unphosphorylated peptide–Mdm2 interactions are of a higher affinity than the unphosphorylated peptide–p300 interactions; this trend reverses when the peptides are phosphorylated. The figure shows that each phosphate moiety engages in ionic interactions, which further stabilize the ATSP_7041 in its bound states.

In contrast to the pThr18_p53_TAD1–Mdm2 simulations, in which the phosphorylation of Thr18 induces conformational changes in the bound peptide, resulting in loss of interactions with Mdm2, no such loss was observed for ATSP_7041 upon phosphorylation of Thr2 (similar to Thr18 in the p53_TAD1 peptide), Tyr6, or Ser12. Our calculations show that the binding energies of ATSP_7041 with single phosphorylation (ATSP_7041^pT2^ or ATSP_7041^pY6^ or ATSP_7041^pS12^) are very similar to the binding of the unphosphorylated peptide (~ –62 kcal/mol and ~ –62 to –68 kcal/mol for the phosphorylated and unphosphorylated peptides, respectively) (Figure 9), but this affinity increases in the case of dual phosphorylation (~ –70 kcal/mol) and when the three residues are phosphorylated (~ –75 kcal/mol) (Figure 9).

**Figure 10:**
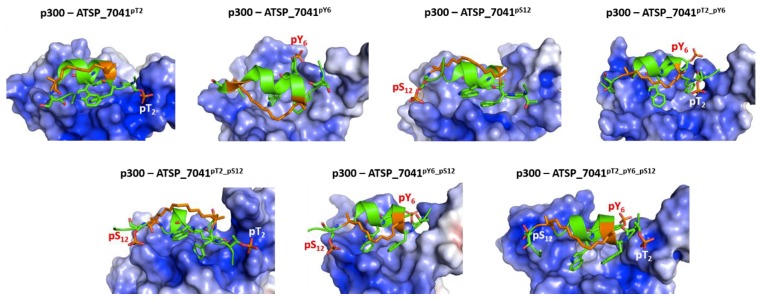
Structural and energetic basis for the binding of the phosphorylated stapled peptide ATSP_7041 peptide and p300. Cartoon/surface representations, based on representative conformations extracted from the MD simulations of the ATSP_7041 peptide phosphorylated at Thr2, Tyr6, and Ser12 bound to p300. The phosphorylated ATSP_7041 peptide is shown as the green cartoon and the p300 is shown as the grey cartoon; p300 is shown with its electrostatic potential surface (red and blue correspond to the –5 kcal/mol and 5 kcal/mol potentials, respectively). It is clear that the highly cationic surface of p300 attracts the anionic surface of the phosphorylated ATSP_7041.

In contrast to their complexes with Mdm2, these peptides undergo increased fluctuations in complex with p300 (Figure S10). This results from the formation of new interactions between the phosphates at pThr2, pTyr6, and pSer12 and the highly positively charged surface of the p300, especially in the region around the peptide binding site (Figure 10) and, in this process, both partners undergo several conformational changes. The bound peptide retains its helical conformation in the regions within the staple, while the peptide C-termini remain unstructured (Figure S11). 

**Figure 11:**
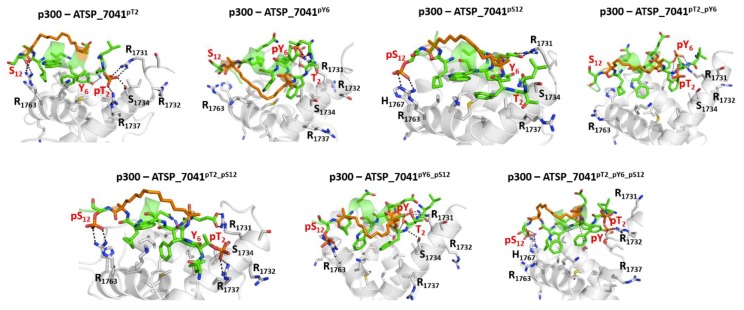
Structural basis for the binding of the phosphorylated stapled peptide ATSP_7041 peptide and p300. Cartoon representations, based on a representative conformations extracted from the MD simulations of the ATSP_7041 peptide phosphorylated at Thr2, Tyr6, and Ser12 bound to p300. The phosphorylated ATSP_7041 peptide is shown as the green cartoon and the p300 is shown as the grey cartoon; residues of the peptides and the binding pocket of p300 are highlighted as sticks and the protein-peptide H-bond interactions are highlighted as dashed lines.

The phosphates on pThr2 and pTyr6 form salt bridges with Arg1731, Arg1732, Ser1734, and Arg137, while the phosphate on pSer12 forms salt bridges with Arg1763 and His1767 (Figure 11). These interactions stabilize the phosphorylated peptides with p300 significantly compared with the unphosphorylated peptide (ranging from ~7 to 14 kcal/mol for single phosphorylation, ~20 kcal/mol for double phosphorylated peptides, and ~50 kcal/mol for the triple phosphorylated peptides; Figure 9).

Thus, it is clear that, although phosphorylation improves the binding of the ATSP_7041 peptides with both Mdm2 and with p300, the improvement in affinity for p300 is 10-fold higher (Figure 9), suggesting that any form of phosphorylation of ATSP_7041 will likely result in competitive binding to p300, and hence attenuate the activity of p53.

### 2.8. Effect of Phosphorylation on the Structural Dynamics of the ATSP_7014 Peptide

We next investigated how the phosphorylation of Thr2, Tyr6, and Ser12 affects the conformations of the free ATSP_7041 peptides in solution. During the biasing potential replica exchange molecular dynamics (BP-REMD) simulations, we see ~49% helicity in the unphosphorylated ATSP_7041^WT^ (Figure S12), with the residues within the staple linkers remaining highly helical (~90% helicity). Introduction of phosphorylation at Thr2 results in a slight increase in helicity (~52%), while that at Tyr6 remains helical for ~50% of the simulation; clearly, neither phosphorylation affects the helicity much. However, phosphorylation of Ser12 perturbs the peptide structure greatly, with only 20% helicity retained including regions within the staple linker. Interestingly, double and triple phosphorylation results in increased flexibility, presumably because of the increased charges, with only 20–30% of helicity retained. Given that, in general, we find that the helical conformation of the peptides is retained upon binding to Mdm2 or to p300, the entropic contribution (the reorganizational energy required for embedding a somewhat disordered peptide in solution to a helical state upon complexation) to the overall binding free energy is expected to be similar for all these peptides; hence, the difference in binding energies observed for these ATSP peptides with Mdm2 and p300 is likely to be mostly enthalpic.

## 3. Discussion

In this work, we investigated the likelihood that ATSP_7041, a peptide inhibitor developed to inhibit Mdm2 and thus activate p53, may have potential non-Mdm2 targets too. We focus our attention on the complex interplay between Mdm2, p53, and the CREB binding protein (CBP)/p300 [34]. It was established that phosphorylation of the disordered TAD domains of p53 [37] is a pre-requisite for its engagement with MDM2 and with transcriptional coactivators such as p300 [28,36,38]. We first establish, using our computational protocol, that phosphorylated p53_TAD1 binds to p300 with a higher affinity than unphosphorylated p53, as shown by experiments [39]. Next, we examine the implications of phosphorylations on a p53 mimicking a peptide inhibitor of Mdm2. A modified version of ATSP_7041 is currently in clinical trials for various indications. ATSP_7041 binds to the p53 binding pocket in the N-terminal domain of Mdm2 by mimicking the interactions of the TAD1 region of p53 with Mdm2. Given that TAD1 engages with the protein p300_Taz2, resulting in the assembly of the transcription machinery, we wondered whether molecules such as ATSP_7041, which mimic TAD1 and bind Mdm2, will also bind p300. If this were true, then ATSP_7041 may potentially also bind p300_Taz2, thus resulting in an attenuation of the amount of free p300 available for interacting with p53. 

Given that the affinity of p53 for p300_Taz2 is increased considerably upon phosphorylation [39], we speculate upon the effects of phosphorylation on three potential phosphorylable sites on ATSP_7041: Thr2, Tyr6, and Ser12. Our simulations suggest that not only is this feasible, but additionally, the phosphorylated ATSP_7041 can bind to p300 with much higher affinity compared with phosphorylated p53 (especially when there are multiple phosphorylations). This is not surprising given that the phosphorylation introduces strong anionicity to the peptide and the surface of p300_Taz2 is highly cationic. It is interesting that phosphorylation of Tyr6 results in the tightest binder of Mdm2 because of the interactions with Lys71 and His74; a high affinity peptide phosphorylated at an equivalent Tyr was also isolated a while back [49].

**Figure 12:**
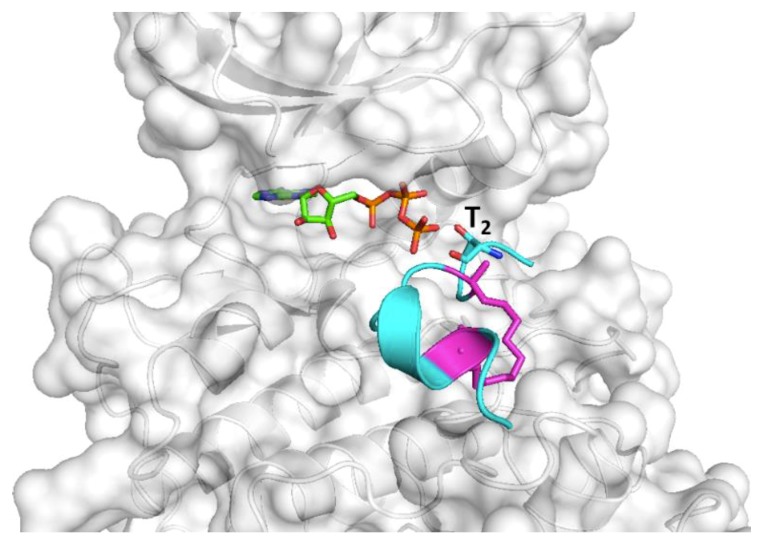
A model of the ATSP_7041 peptide bound with a serine/threonine kinase CK1-δ (generated using the crystal structure of CDK2 bound with a substrate, PDB: 1QMZ; CK1-δ and CDK2 share ~46% homology within their kinase domains). The kinase is shown as surface (grey), the bound peptide shown as the cartoon (cyan) with the staple linker (magenta), and Thr2 is highlighted as sticks. The bound ATP is shown as sticks (green).

In conclusion, our calculations show that, while ATSP_7041 binds to Mdm2 tighter than to p300_Taz2, if Thr2 and/or Tyr6 and/or Ser12 in ATSP_7041 were to be phosphorylated, its affinity for p300_Taz2 would be significant, and hence reduce its ability to fully stabilize p53; in house experimental data (to be published elsewhere) appears to at least partially concur with this hypothesis. Future work could explore variants of such peptides to exploit the observation that multiple phosphorylations of the TAD1 domain of p53 can elicit increased engagements with the coactivators [27,36,38]. Recent data on alanine mutants of ATSP_7041 [50] show that an Ala substitution at the Thr2, Tyr6, and Ser12 retain binding to Mdm2 without losing the ability to activate p53 in cells. We can assume that these mutants do not get phosphorylated and may not bind p300. This suggests that appropriately designed mutations at these positions may be harnessed to inhibit Mdm2, but reduce or disable binding to p300. Finally, we wondered whether the conformational constraints of the stapled peptide ATSP_7041 would enable it to be phosphorylated. To explore this, we constructed a model of ATSP_7041 docked to serine/threonine kinase, which was shown to phosphorylate p53 at Thr18 (equivalent position in ATSP_7041 is Thr2). For the docking, we extracted a conformation of ATSP_7041 from its apo simulations. It is clear from the docked conformation (Figure 12) that ATSP_7041 can easily dock onto the substrate binding site of CK2, with its Thr2 side chain hydroxyl appropriately positioned to accept a phosphate from the bound ATP. Overall, this study highlights the importance of assessing the off-target effects of peptide inhibitors, particularly guided by the understanding of the networks of PPIs that are being targeted.

## 4. Materials and Methods

### 4.1. Model Preparation

For the simulations of the peptides bound to the N-terminal domain of Mdm2, the available crystal structure of p53 peptide (residues 17 to 29) (ETFSDLWKLLPEN) bound to the N-terminal domain (residues 25 to 109) of Mdm2 (PDB: 1YCR) and a model of the structure of ATSP_7041 peptide bound to the N-terminal domain of Mdm2 generated recently [50] were used. For the simulation of the structures of the complex between the peptides and p300_Taz2, the structure of truncated p53 peptide (residues from 17 to 29) bound with Taz2 domain of p300 was generated from the solution structure of a long segment of p53 (residues 1 to 39) bound to the Taz2 domain (residues 1723 to 1812) of p300 (PDB: 2K8F). The model of ATSP_7041 bound to p300_Taz2 was generated using the structure of p53 peptide bound to p300_Taz2. The complexes of the phosphorylated peptides bound to Mdm2 or to p300_Taz2 were generated from the structures of the complexes of the unphosphorylated peptides with Mdm2 and with p300_Taz2. For the simulations of the apo peptides in solution, the peptides in their extended state conformations were generated in the *xleap* module of the program Amber 18 [51].

### 4.2. Molecular Dynamics (MD) Simulations

MD simulations were carried out on the free apo peptides and on the different complexes generated above. The *xleap* module of the Amber18 program [51] was used to prepare the system for the MD simulations. The N- and C-termini of the peptides were capped with the acetyl (ACE) and amide (NHE) moieties, respectively. The parameters for the staple linkers were taken from our previous study [52] and parameters for phosphorylated serine/threonine/tyrosine were taken as described elsewhere [53]. All simulation systems were neutralized with an appropriate number of counter ions. The neutralized systems were each solvated in an octahedral box with TIP3P [54] water molecules, leaving at least 10 Ǻ between the solute atoms and the borders of the box. MD simulations were carried out with the pmemd. CUDA module of the Amber18 program in combination with the ff14SB force field [55]. All MD simulations were carried out in explicit solvent at 300 K. During all the simulations, the long-range electrostatic interactions were treated with the particle mesh Ewald [56] method, using a real space cut off distance of 9 Å. The SETTLE [57] algorithm was used to constrain bond vibrations involving hydrogen atoms, which allowed a time step of 2 fs during the simulations. Solvent molecules and counter ions were initially relaxed using energy minimization with restraints on the protein and peptide atoms. This was followed by unrestrained energy minimization to remove any steric clashes. Subsequently, the system was gradually heated from 0 to 300 K using MD simulations with positional restraints (force constant: 50 kcal mol^-1^ Å^-2^) on the atoms of the protein and peptides over a period of 0.25 ns, allowing water molecules and ions to move freely, followed by gradual removal of the positional restraints and a 2 ns unrestrained equilibration at 300 K. The resulting systems were used as starting structures for the respective production phase of the MD simulations. For each case, three independent (using different initial random velocities) MD simulations were carried out for 250 ns starting from the well equilibrated structures. To enhance the conformational sampling, each of these peptides were subjected to biasing potential replica exchange MD (BP-REMD) simulations. The BP-REMD technique is a type of Hamiltonian-REMD method, which includes a biasing potential that promotes dihedral transitions along the replicas [58,59]. For each system, BP-REMD was carried out with eight replicas including a reference replica without any bias. BP-REMD was carried out for 100 ns with the exchange between the neighbouring replicas attempted every 2 ps and accepted or rejected according to the metropolis criteria. Conformations sampled at the reference replica (no bias) were used for further analysis. Simulation trajectories were visualized using visual molecular dynamics (VMDs) [60] and figures were generated using Pymol [61].

### 4.3. Binding Energy Calculations and Energy Decomposition Analysis

Molecular mechanics Poisson Boltzmann surface area (MMPBSA) methods were used for the calculation of binding free energies between the peptides and their partner proteins [62,63]. Binding energy calculations were carried out with a salt concentration of 150 mM using 250 conformations extracted from the last 100 ns of the simulations. Entropy calculations are computationally intensive and do not converge easily, and hence are ignored. The effective binding energies were decomposed into contributions of individual residues using the molecular mechanics generalized boran surface area (MMGBSA) energy decomposition scheme. The MMGBSA calculations were carried out using the same protocol as in the MMPBSA calculations. The polar contribution to the solvation free energy was determined by applying the generalized born (GB) method (igb = 2) [51], using mbondi2 radii. The non-polar contributions were estimated using the ICOSA method [51] by a solvent accessible surface area (SASA) dependent term scaled with a surface tension proportionality constant of 0.0072 kcal/mol Å^2^.

## 5. Conclusions

Protein-protein interactions are central to most biological processes, and hence their dysregulation results in disease. Approximately 40% of all PPIs are mediated by relatively short peptide segments, and these are often intrinsically disordered (IDR), thus enabling each segment to engage in multiple PPIs. These short peptide motifs can be mimicked and offer the possibility of developing peptide-based inhibitors to inhibit PPIs. These interfaces are often flat, and hence not druggable by small molecules; therefore, the possibility of developing peptidomimetics is quite appealing, expanding access to this vast untapped reservoir of “undruggable targets”. Peptide-based drugs are increasingly finding applications as therapeutics, with ~60 drugs approved by the FDA for various indications and several more in clinical development. Given that the parent/native peptide is involved in multiple PPIs, peptidic inhibitors that are developed from the native peptide may interfere with multiple PPIs, resulting in reduced efficacy and also off-target effects resulting in toxicity. We explored the likelihood of one such peptidic inhibitor (the p53 activator ATSP_7041), targeted at inhibiting Mdm2, which can, upon post-translational modifications, bind to at least one other protein (p300), thereby resulting in reduced efficacy of the peptide. Our findings suggest that careful consideration of specificity has to be taken into account while designing peptide inhibitors.

## Supplementary Materials

Supplementary materials can be found at https://www.mdpi.com/1422-0067/20/23/5996/s1. Figure S1. Structural basis for the binding of p53_TAD1 peptide with p300. Figure S2. Cartoon representation, based on a representative conformation extracted from the MD simulations, of the p53_TAD1 peptide bound to p300. Figure S3. Estimation of the free energies (ΔG) of the interactions between the p53 TAD1 peptides with Mdm2 or p300. Figure S4. The structural changes that occur in the phosphorylated p53_TAD1 peptide with p300 during the MD simulations. Figure S5. The structural changes that occur in the phosphorylated p53_TAD1 peptide with Mdm2 during the MD simulations. Figure S6. The structural changes that occur in the unphosphorylated and phosphorylated p53_TAD1 peptide in solution during the MD simulations. Figure S7. Estimation of the free energies (ΔG) of the interactions between the ATSP_7041 peptide with Mdm2 or p300. Figure S8. The structural changes that occur in the phosphorylated ATSP_7041 peptides with Mdm2 during the MD simulations of the complexes as measured by RMSD. Figure S9. The structural changes that occur in the phosphorylated ATSP_7041 peptides with Mdm2 during the MD simulations. Figure S10. The structural changes that occur in the phosphorylated ATSP_7041 peptides with p300 during the MD simulations of the complexesas measured by RMSD Figure S11. The structural changes that occur in the phosphorylated ATSP_7041 peptides with p300 during the MD simulations of the complexes. Figure S12. The structural changes that occur in the unphosphorylated and phosphorylated ATSP_7041 peptides in solution during the MD simulations.
