# Supplementary material for: The Dual Interactions of p53 with MDM2 and p300: Implications for the Design of MDM2 Inhibitors
Source: Int J Mol Sci. 2019 Nov 28;20(23):5996. doi: 10.3390/ijms20235996 (PMC6928821; doi:10.3390/ijms20235996)
Supplement: Supplementary file 1 [file ijms-20-05996-s001.pdf]

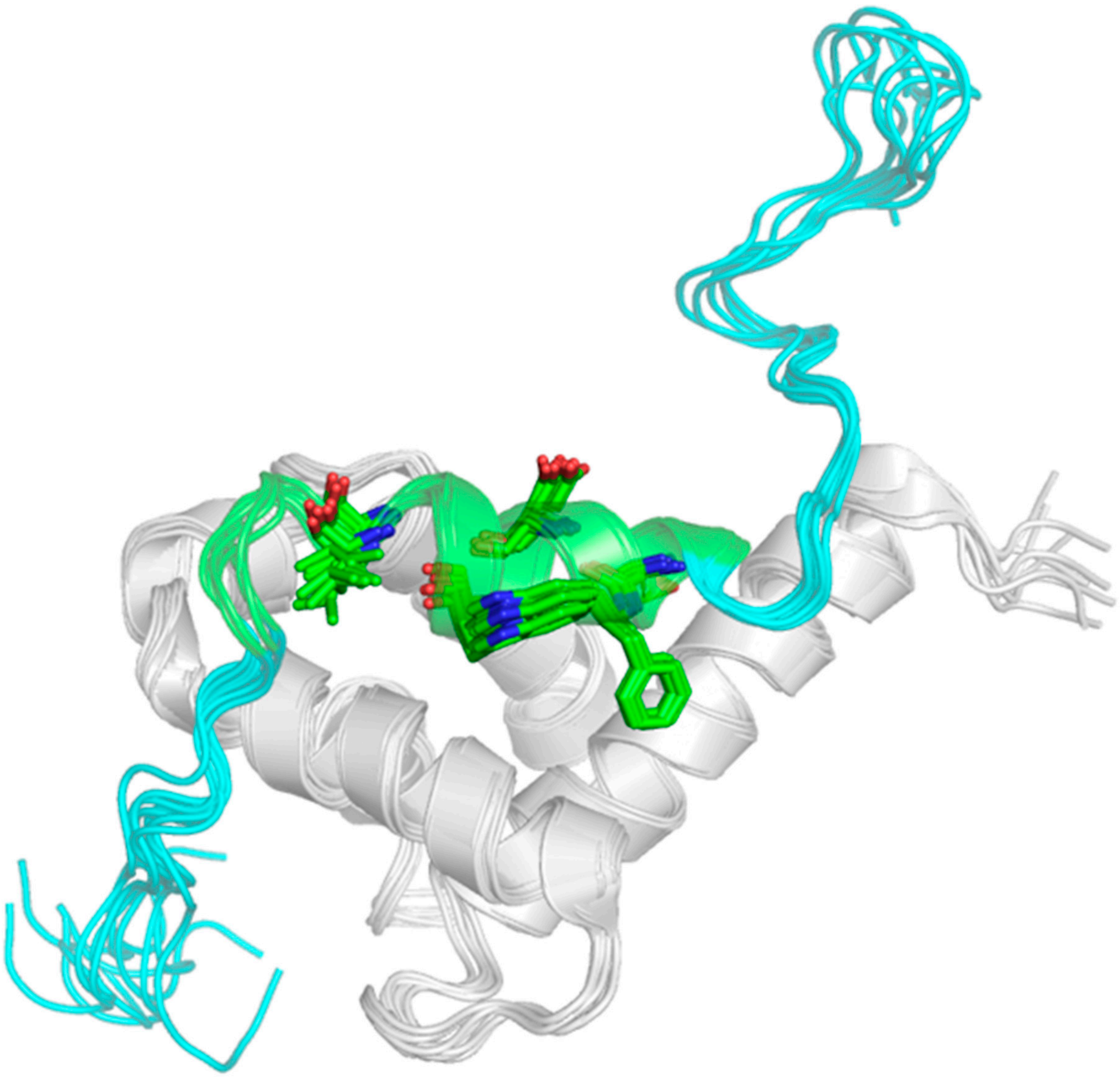

Figure S1. Structural basis for the binding of p53_TAD1 peptide with p300. Cartoon representation of the NMR ensemble, depicting how the p53_TAD1 (residues 1 to 39) bound to p300 protein (residues 1723 to 1812). The p53_TAD1 peptide is shown as the green cartoon (disordered regions in cyan colour) with the p300 protein shown as the grey cartoon.

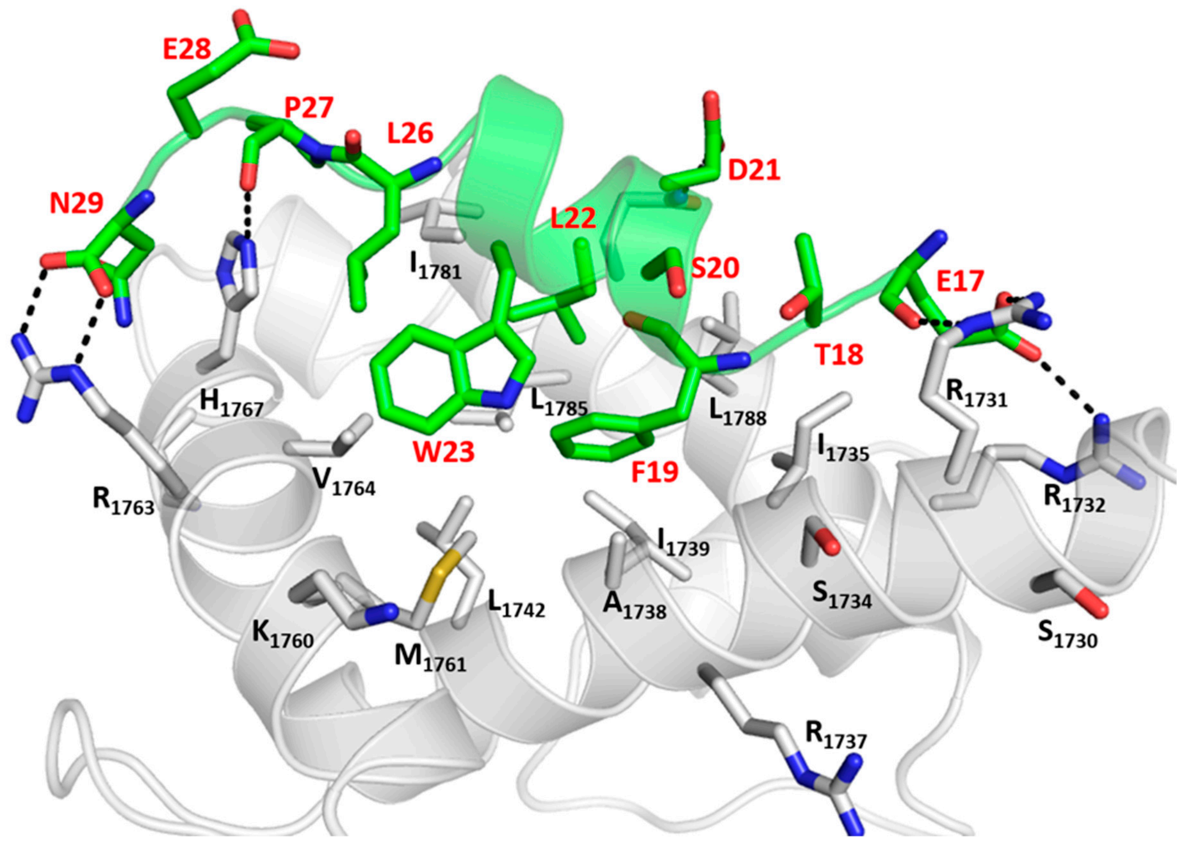

Figure S2. Structural basis for the binding of the p53_TAD1 peptide with p300. Cartoon representation, based on a representative conformation extracted from the MD simulations, of the p53_TAD1 peptide bound to p300. The p53_TAD1 peptide is shown as the green cartoon and the protein is shown as the grey cartoon; residues of the peptides and the binding pocket of p300 are highlighted as sticks and the protein–peptide H-bond interactions are highlighted as dashed lines.

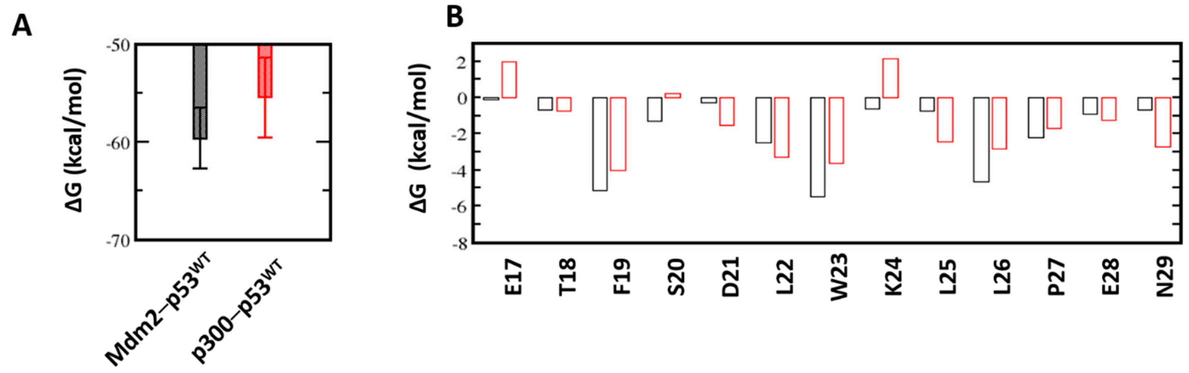

Figure S3. (**A**) Estimation of the free energies (ΔG) of the interactions between the p53 TAD1 peptides with Mdm2 or p300, using the MMPBSA approximations from the conformations generated from MD simulations of the complexes. Higher affinities are reflected by larger negative values; it is clear that the p53_TAD1 peptide–Mdm2 interactions are of a higher affinity than the p53_TAD1 peptide–p300 interactions. (**B**) Estimation of the contribution of individual residues to the net binding energy between p53_TAD1 with Mdm2 or p300, using the molecular mechanics generalized boran surface area (MMGBSA) approximations from the conformations generated from MD simulations of the complexes.

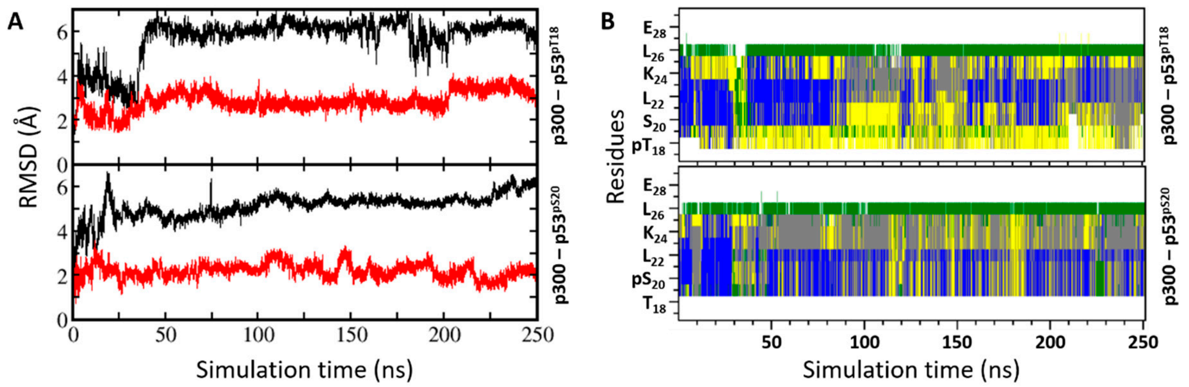

Figure S4. The structural changes that occur in the phosphorylated p53_TAD1 peptide with p300 during the MD simulations of the complexes as measured by RMSD. The RMSD of the p53_TAD1 peptide phosphorylated at Thr18 (top of panel **A**), phosphorylated at Ser20 (bottom of panel **A**) (calculated against the starting conformation of the MD simulations) is shown in red; the RMSD of the p300 protein in complex with p53_TAD1 peptide phosphorylated at Thr18 (top of panel **A**), phosphorylated at Ser20 (bottom of panel **A**) (calculated against the starting conformation of the MD simulations) is shown in black. Panel B shows the changes in the secondary structures of the p53_TAD1 peptide phosphorylated at Thr18 (top of panel **B**), phosphorylated at Ser20 (bottom of panel **B**) when bound to p300; the secondary structures were calculated using the DSSP program and are shown as follows: blue for α-helix, grey for 3_{10}-helix, yellow for turn, green for bend, and white for coil, along the peptide chain (y-axis) as a function of the simulation time (x-axis).

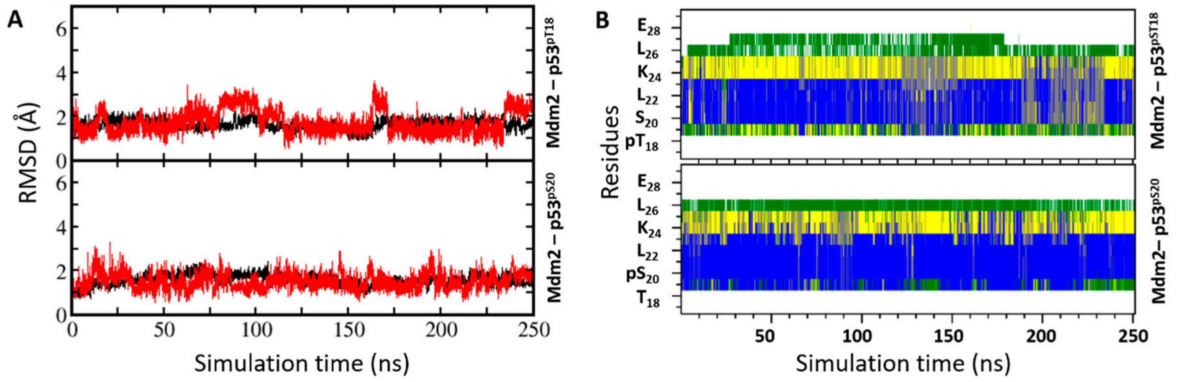

Figure S5. The structural changes that occur in the phosphorylated p53_TAD1 peptide with Mdm2 during the MD simulations of the complexes as measured by RMSD. The RMSD of the p53_TAD1 peptide phosphorylated at Thr18 (top of panel **A**), phosphorylated at Ser20 (bottom of panel **A**) (calculated against the starting conformation of the MD simulations) is shown in red; the RMSD of the Mdm2 protein in complex with p53_TAD1 peptide phosphorylated at Thr18 (top of panel **A**), phosphorylated at Ser20 (bottom of panel **A**) (calculated against the starting conformation of the MD simulations) is shown in black. Panel B shows the changes in the secondary structures of the p53_TAD1 peptide phosphorylated at Thr18 (top of panel **B**), phosphorylated at Ser20 (bottom of panel **B**) when bound to Mdm2; the secondary structures were calculated using the DSSP program and are shown as follows: blue for α-helix, grey for 3_{10}-helix, yellow for turn, green for bend, and white for coil, along the peptide chain (y-axis) as a function of the simulation time (x-axis).

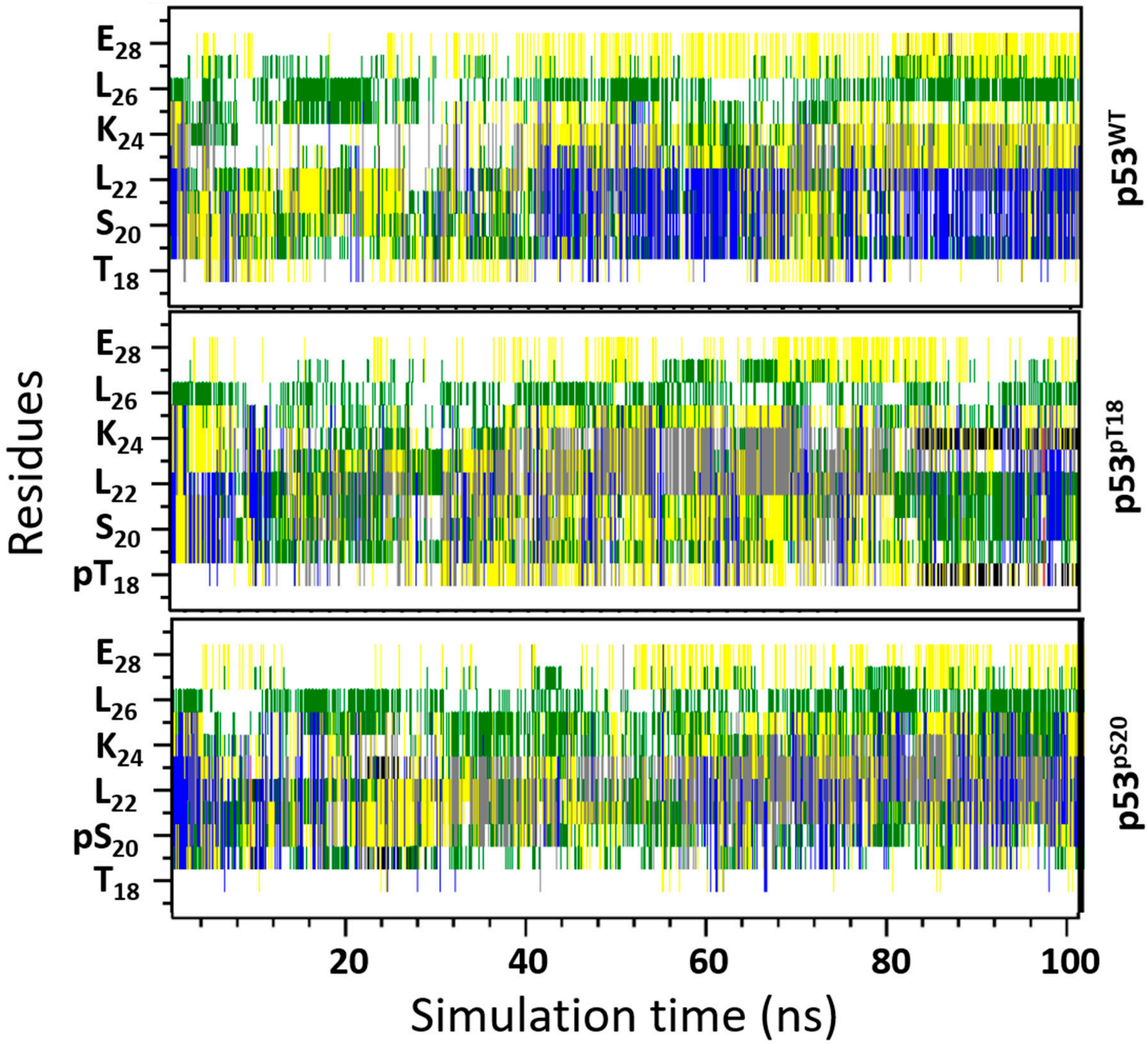

Figure S6. The structural changes that occur in the unphosphorylated and phosphorylated p53_TAD1 peptide in solution during the MD simulations. The panel show the changes in the secondary structures of the p53_TAD1 peptide unphosphorylated (top) and phosphorylated at Thr18 (middle), phosphorylated at Ser20 (bottom) in solution; the secondary structures were calculated using the DSSP program and are shown as follows: blue for α-helix, grey for 3_{10}-helix, yellow for turn, green for bend, and white for coil, along the peptide chain (y-axis) as a function of the simulation time (x-axis).

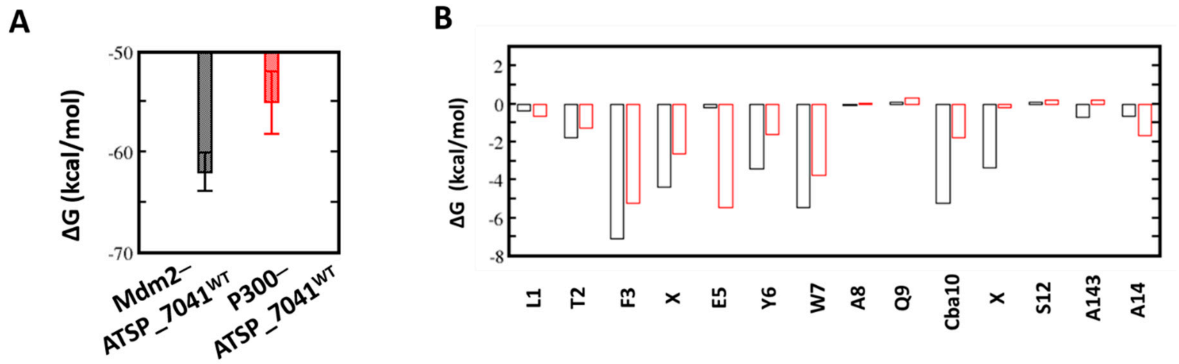

Figure S7. (**A**) Estimation of the free energies (ΔG) of the interactions between the ATSP_7041 peptide with Mdm2 or p300, using the MMPBSA approximations from the conformations generated from MD simulations

of the complexes. Higher affinities are reflected by larger negative values; it is clear that the ATSP_7041 peptide–Mdm2 interactions are of a higher affinity than the ATSP_7041 peptide–p300 interactions. (**B**) Estimation of the contribution of individual residues to the net binding energy between ATSP_7041 with Mdm2 or p300, using the MMGBSA approximations from the conformations generated from MD simulations of the complexes.

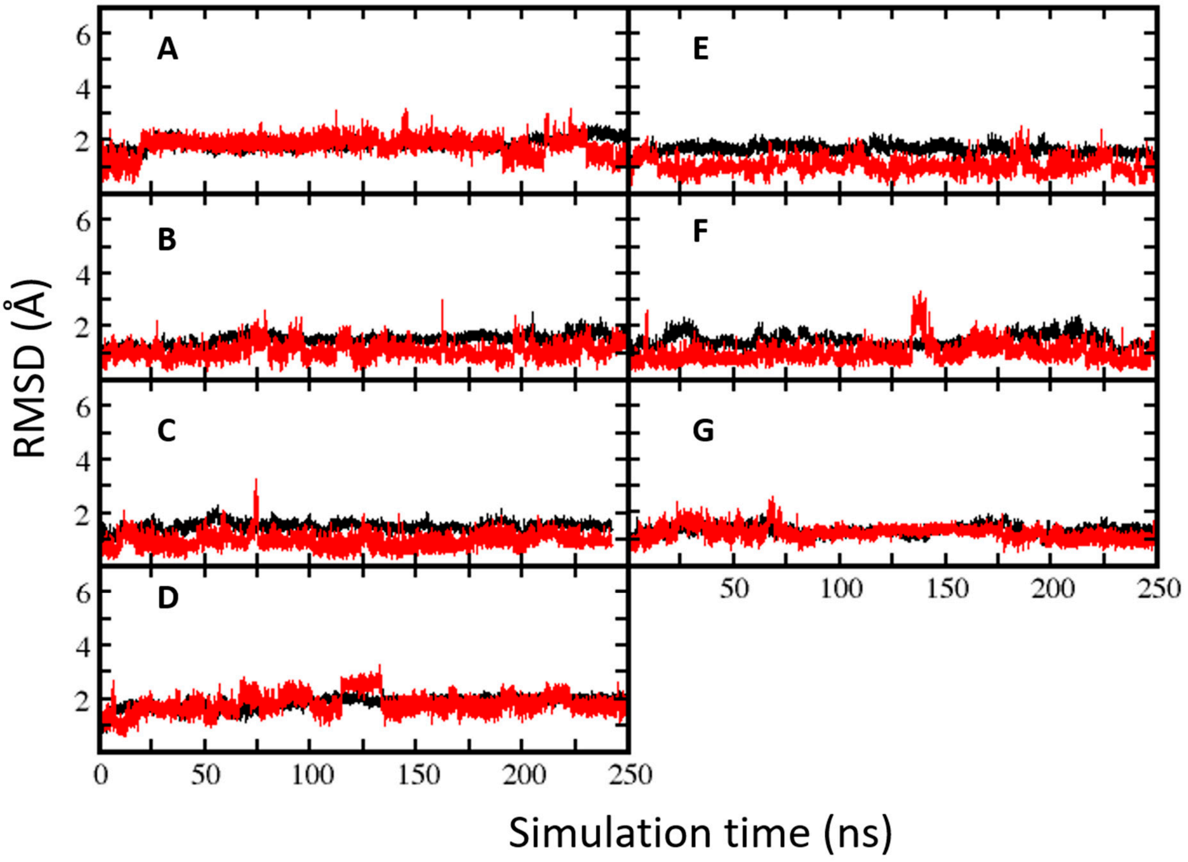

Figure S8. The structural changes that occur in the phosphorylated ATSP_7041 peptides with Mdm2 during the MD simulations of the complexes as measured by RMSD. The RMSD of the phosphorylated ATSP_7041 peptides (**A**) ATSP_7041^{pT2}; (**B**) ATSP_7041^{pY6}; (**C**) ATSP_7041^{pS12}; (**D**) ATSP_7041^{pT2_pY6}; (**E**) ATSP_7041^{pT2_pS12}; (**F**) ATSP_7041^{pY6_pS12}; and (**G**) ATSP_7041^{pT2_pY6_pS12} (calculated against the starting conformation of the MD simulations) is shown in red for the Mdm2 complex. The RMSD of the corresponding peptide bound Mdm2 protein (calculated against the starting conformation of the MD simulations) is shown in black.

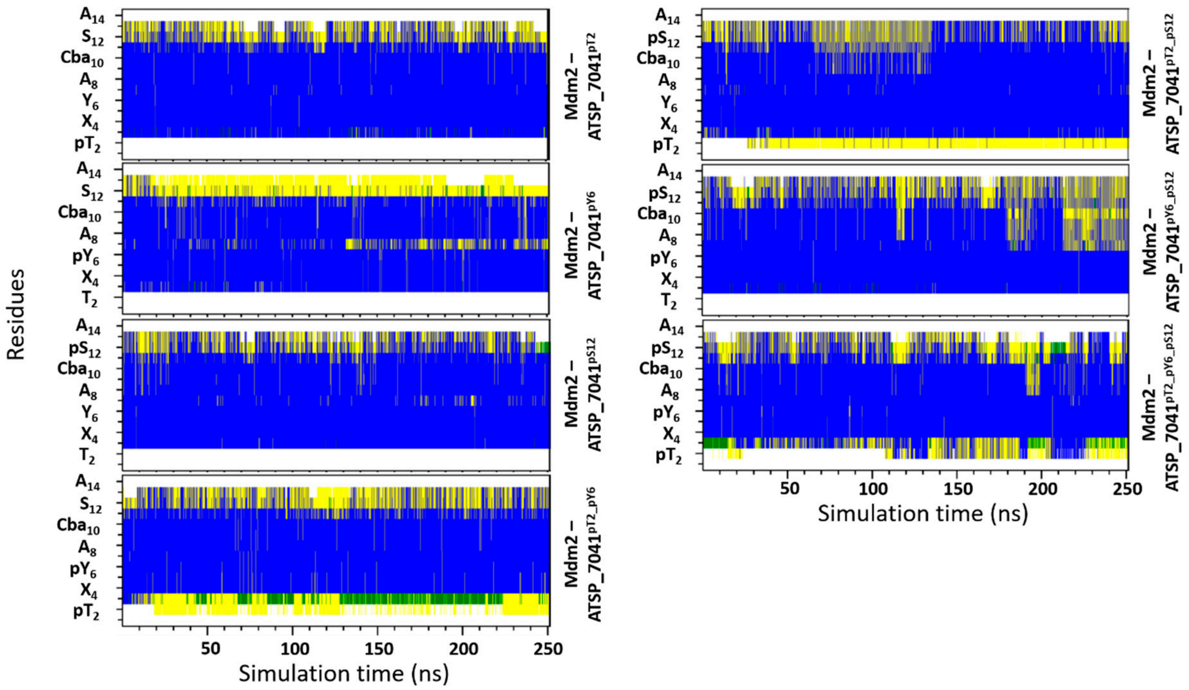

Figure S9. The structural changes that occur in the phosphorylated ATSP_7041 peptides with Mdm2 during the MD simulations of the complexes. The panel show the changes in the secondary structures of the phosphorylated ATSP-7041 peptides (ATSP_7041^{pT2}; ATSP_7041^{pY6}; ATSP_7041^{pS12}; ATSP_7041^{pT2_pY6}; ATSP_7041^{pT2_pS12}; ATSP_7041^{pY6_pS12}; and ATSP_7041^{pT2_pY6_pS12}); the secondary structures were calculated using the DSSP program and are shown as follows: blue for α-helix, grey for 3_{10}-helix, yellow for turn, green for bend, and white for coil, along the peptide chain (y-axis) as a function of the simulation time (x-axis).

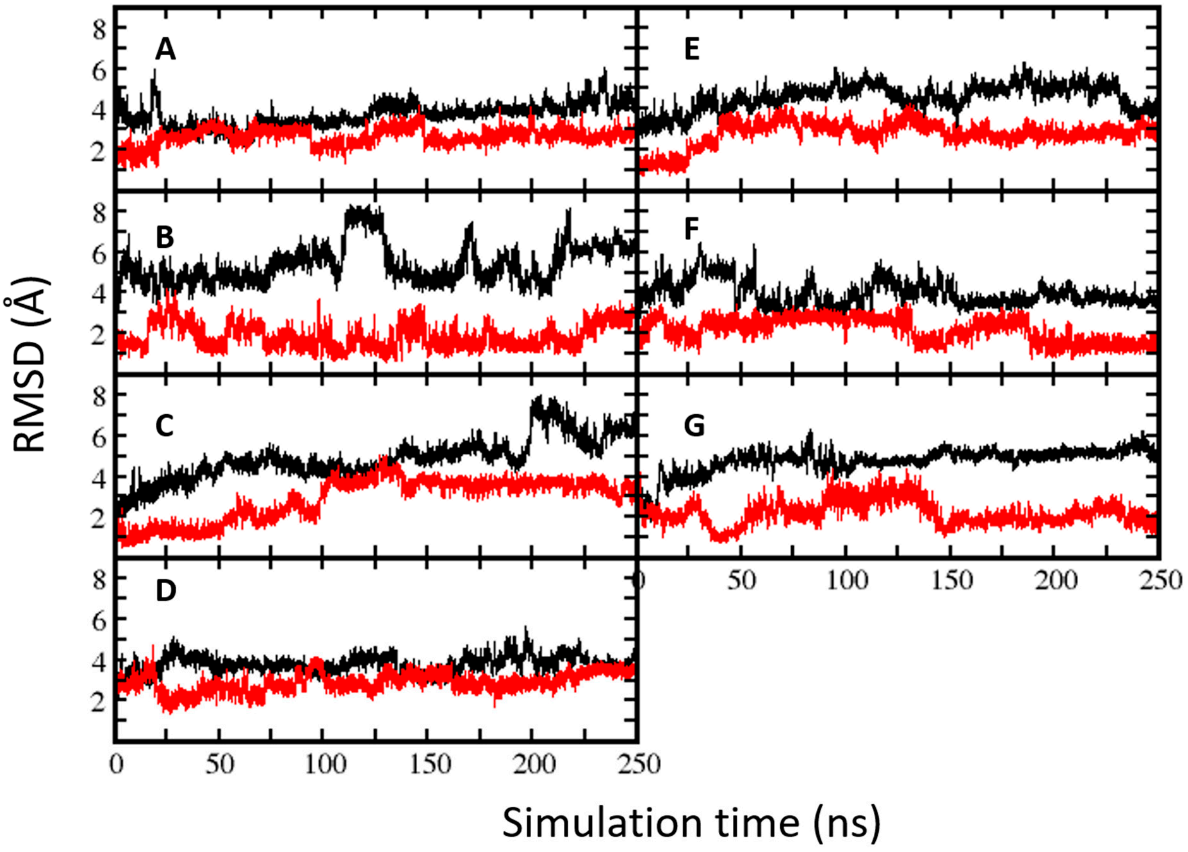

Figure S10. The structural changes that occur in the phosphorylated ATSP_7041 peptides with p300 during the MD simulations of the complexes as measured by RMSD. The RMSD of the phosphorylated ATSP_7041 peptides (**A**) ATSP_7041^{pT2}; (**B**) ATSP_7041^{pY6}; (**C**) ATSP_7041^{pS12}; (**D**) ATSP_7041^{pT2_pY6}; (**E**) ATSP_7041^{pT2_pS12}; (**F**) ATSP_7041^{pY6_pS12}; and (**G**) ATSP_7041^{pT2_pY6_pS12} (calculated against the starting conformation of the MD simulations) is shown in red for the p300 complex; and the RMSD of the corresponding peptide bound p300 protein (calculated against the starting conformation of the MD simulations) is shown in black.

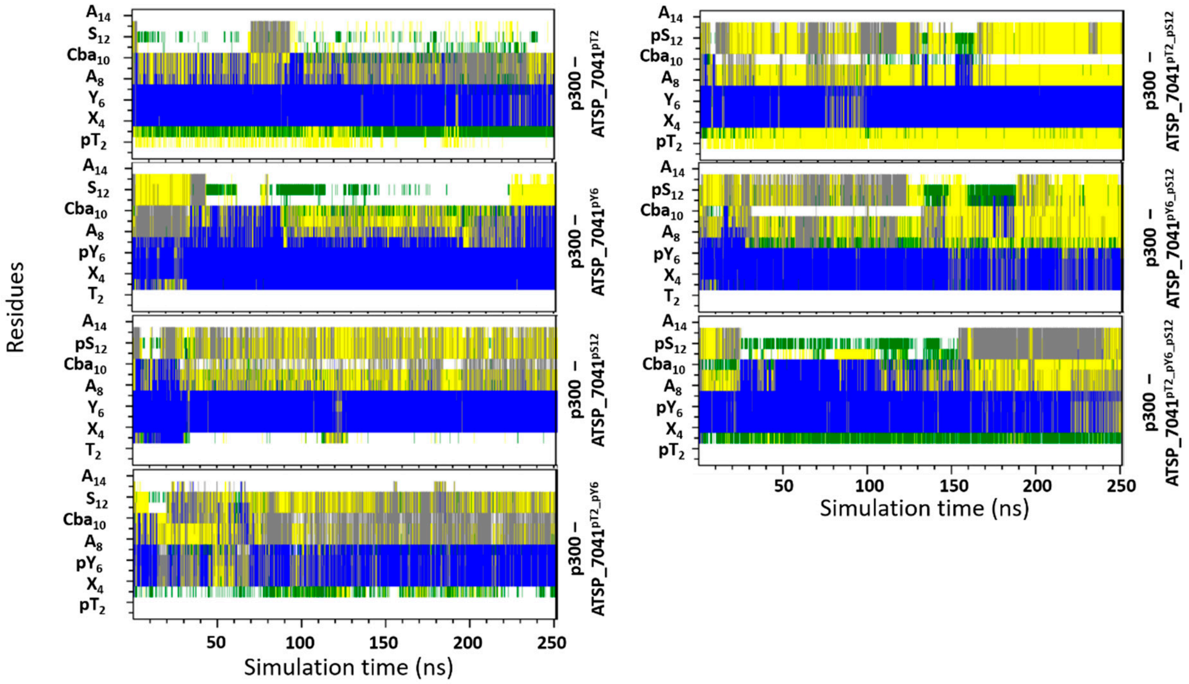

Figure S11. The structural changes that occur in the phosphorylated ATSP_7041 peptides with p300 during the MD simulations of the complexes. The panel show the changes in the secondary structures of the phosphorylated ATSP_7041 peptides (ATSP_7041^{pT2}; ATSP_7041^{pY6}; ATSP_7041^{pS12}; ATSP_7041^{pT2_pY6}; ATSP_7041^{pT2_pS12}; ATSP_7041^{pY6_pS12}; and ATSP_7041^{pT2_pY6_pS12}); the secondary structures were calculated using the DSSP program and are shown as follows: blue for α-helix, grey for 3_{10}-helix, yellow for turn, green for bend, and white for coil, along the peptide chain (y-axis) as a function of the simulation time (x-axis).

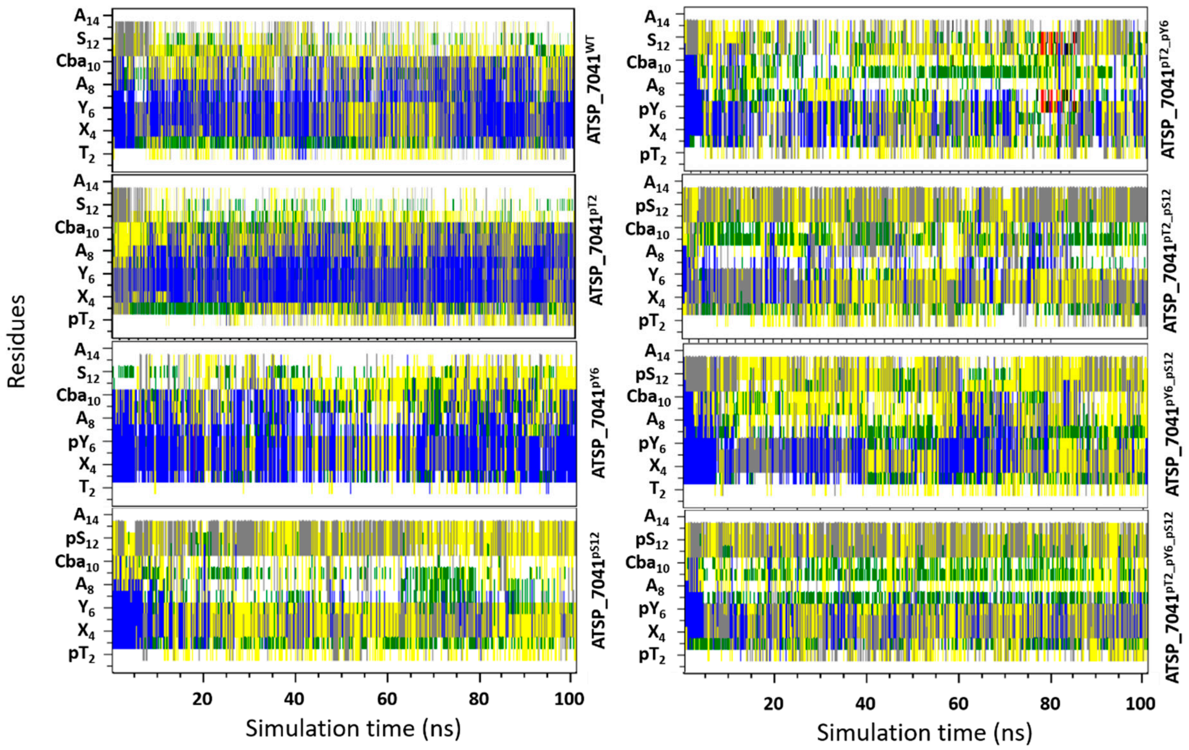

Figure S12. The structural changes that occur in the unphosphorylated and phosphorylated ATSP_7041 peptides in solution during the MD simulations. The panel shows the changes in the secondary structures of the unphosphorylated and phosphorylated ATSP_7041 peptides (ATSP_7041^{WT}; ATSP_7041^{pT2}; ATSP_7041^{pY6}; ATSP_7041^{pS12}; ATSP_7041^{pT2_pY6}; ATSP_7041^{pT2_pS12}; ATSP_7041^{pY6_pS12}; and ATSP_7041^{pT2_pY6_pS12}); the secondary structures were calculated using the DSSP program and are shown as follows: blue for α-helix, grey for 3_{10}-helix, yellow for turn, green for bend, and white for coil, along the peptide chain (y-axis) as a function of the simulation time (x-axis).
